# Electrospun Nanofiber-Based Ceramic Aerogels: Synergistic Strategies for Design and Functionalization

**DOI:** 10.1007/s40820-025-01864-4

**Published:** 2025-08-06

**Authors:** Panpan Li, Xuan Zhang, Ying Li, Cunyi Zhao, Jianyong Yu, Yang Si

**Affiliations:** 1 State Key Laboratory of Advanced Fiber Materials, College of Textiles, Donghua University, Shanghai, 201620 People’s Republic of China; 2 College of Materials Science and Engineering, Donghua University, Shanghai, 201620 People’s Republic of China; 3 Innovation Center for Textile Science and Technology, Donghua University, Shanghai, 200051 People’s Republic of China

**Keywords:** Electrospinning nanofibers, Ceramic aerogels, Mechanical properties

## Abstract

Ceramic aerogels (CAs) have emerged as a significant research frontier across various applications due to their lightweight, high porosity, and easily tunable structural characteristics. However, the intrinsic weak interactions among the constituent nanoparticles, coupled with the limited toughness of traditional CAs, make them susceptible to structural collapse or even catastrophic failure when exposed to complex mechanical external forces. Unlike 0D building units, 1D ceramic nanofibers (CNFs) possess a high aspect ratio and exceptional flexibility simultaneously, which are desirable building blocks for elastic CAs. This review presents the recent progress in electrospun ceramic nanofibrous aerogels (ECNFAs) that are constructed using ECNFs as building blocks, focusing on the various preparation methods and corresponding structural characteristics, strategies for optimizing mechanical performance, and a wide range of applications. The methods for preparing ECNFs and ECNFAs with diverse structures were initially explored, followed by the implementation of optimization strategies for enhancing ECNFAs, emphasizing the improvement of reinforcing the ECNFs, establishing the bonding effects between ECNFs, and designing the aggregate structures of the aerogels. Moreover, the applications of ECNFAs across various fields are also discussed. Finally, it highlights the existing challenges and potential opportunities for ECNFAs to achieve superior properties and realize promising prospects.

## Highlights

- This review provides comprehensive fabrication methods for the manufacturing of electrospun ceramic nanofibrous aerogels and offers professional guidance for materials development in this field.
- The optimization strategies for electrospun ceramic nanofibrous aerogels (ECNFAs)’ mechanical properties have been provided, highlighting multi-scale design from nano-building blocks to nanofiber aggregate structure design.
- This review systematically introduces the diverse roles of ECNFAs in specific application scenarios and application-specific mechanisms and provides transformative solutions for advanced engineering applications.

## Graphical abstract

**Figure:**
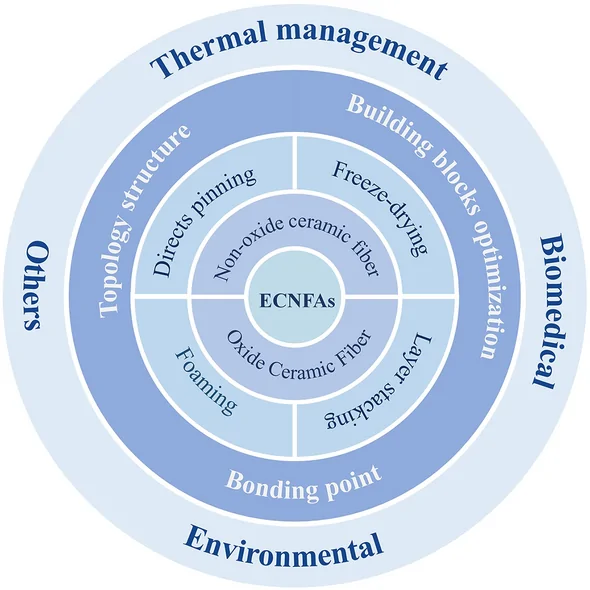

## Introduction

Aerogel was initially proposed by Kistler in 1931 [1], which was fabricated by replacing the liquid in an inorganic gel with gas while maintaining the solid network structure of the gel without shrinkage through supercritical drying techniques [2]. Due to the continuity of the displaced solution within the sol, the resulting aerogels contain over 80% air, which renders them among the lightest solid materials known to date. The aerogel was ranked among the top ten emerging technologies in chemistry for 2022 by the International Union of Pure and Applied Chemistry (IUPAC) due to its distinctive characteristics, such as an extraordinarily high porosity, an extremely low bulk density of 0.1 mg cm^−3^ [3], and a low thermal conductivity to 0.012 W m^−1^ K^−1^ [4]. These unique characteristics also make aerogels highly promising for a wide range of applications, such as aerospace, environmental science, medicine, and other fields [5]. Among the various types of aerogels (polymer aerogels, carbon aerogels, metal aerogels, and ceramic aerogels), ceramic aerogels are distinguished by their exceptional heat resistance, high-temperature oxidation resistance, and corrosion resistance, exhibiting better service performance in complex extreme environments than other aerogels. Regrettably, ceramic aerogels demonstrate poor mechanical properties and may fracture into small fragments when subjected to external forces. The deficiency can be attributed to the necklace-like arrangement of nanoparticle chains within their frameworks, which results from the limited condensation of sol particles during the sol–gel process [4]. Recent studies have demonstrated that the utilization of 1D continuous and flexible ceramic nanofibers (CNFs) as building blocks can effectively address the inherent brittleness of ceramic aerogels (CAs) by avoiding the weak neck connections effect [6].

The fabrication of CNFs employs diverse methodologies including molecular self-assembly [7, 8], electrospinning [9–13], blow spinning [14–24], centrifugal spinning [25–27], and dry spinning [28, 29], wherein the electrospinning has emerged as the predominant method due to its capacity to generate continuous CNFs with tunable surface properties, customizable compositions, and controllable structural configurations. Recent advancements have demonstrated a novel approach for creating elastic CAs through freeze-drying-assisted self-assembly of electrospun ceramic nanofibrous (ECNFs) into three-dimensional elastic frameworks. The method enables electrospun ceramic nanofibrous aerogels (ECNFAs) to rapidly recover their original state following the release of stress [6], which mainly arises from a multi-scale structural hierarchy: at the microscale, ECNFs exhibit sufficient flexibility and modulus to absorb and dissipate impact energy, ensuring that the nanofibers remain intact without breaking; at the mesoscale, there is sufficient bonding between the nanofibers to maintain the stability of the fiber network under external forces; at the macroscale, the network structure formed by self-assembled nanofibers effectively dissipates stress through structural deformation. The outstanding mechanical properties of ECNFAs, along with their distinctive acoustic, optical, electrical, thermal, and magnetic characteristics, have further broadened their range of applications. Meanwhile, the emerging material has garnered significant interest [30], bringing about considerable developments in the field of ECNFAs. For example, the types of ECNFAs have expanded from the single Si series to other series including Zr, Al, and Ti. Regrettably, although numerous articles have investigated ECNFAs, there remains a lack of comprehensive reviews on this field.

**Fig. 1:**
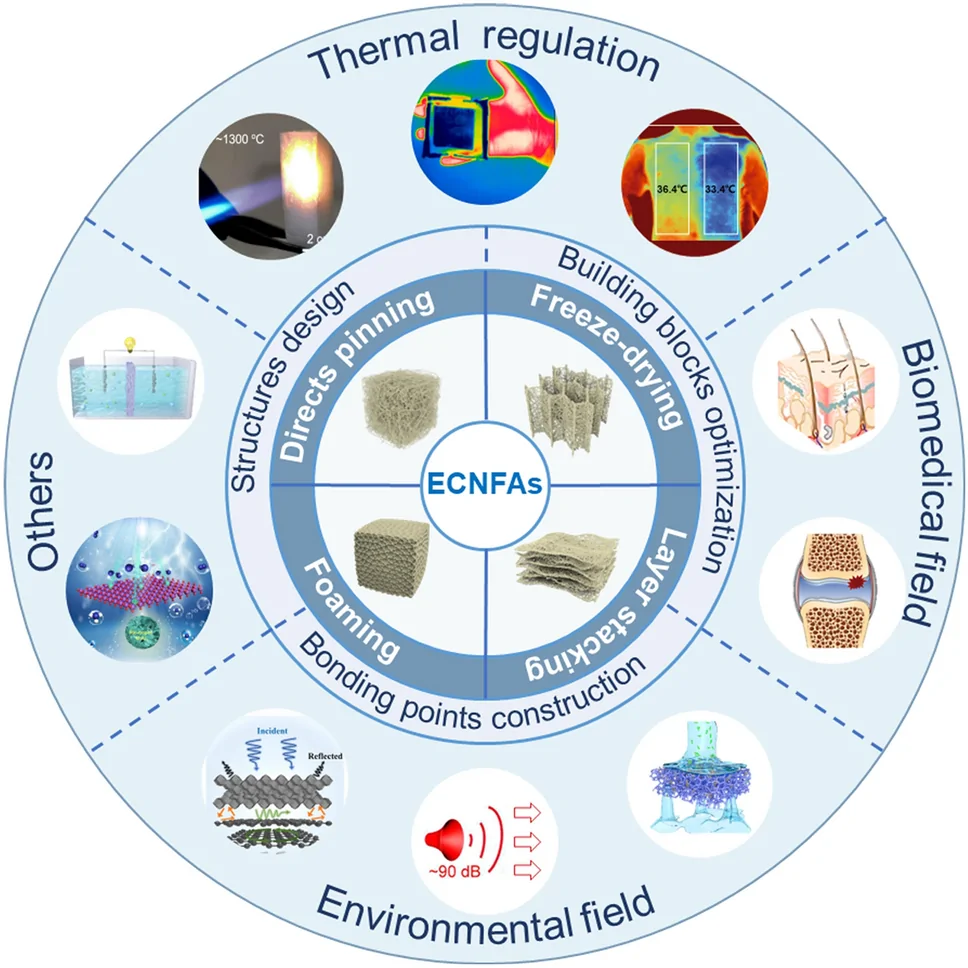
The applications of ECNFAs derived from various methods and featuring different structures across multiple fields. Distinct ECNFAs are utilized in thermal management [79, 129, 280], environmental applications [73, 161, 281], biomedical contexts [282, 283], and other areas [251, 284]. Adapted with permission from Ref. [79]. Copyright 2022, Springer Nature. Adapted with permission from Ref. [280]. Copyright 2018, American Chemical Society. Adapted with permission from Ref. [129]. Copyright 2021, American Association for the Advancement of Science. Adapted with permission from Ref. [161]. Copyright 2020, American Chemical Society. Adapted with permission from Ref. [73]. Copyright 2022, American Chemical Society. Adapted with permission from Ref. [281]. Copyright 2022, Elsevier. Adapted with permission from Ref. [282]. Copyright 2019, Wiley–VCH. Adapted with permission from Ref. [283]. Copyright 2023, Elsevier. Adapted with permission from Ref. [251]. Copyright 2023, Wiley–VCH. Adapted with permission from Ref. [284]. Copyright 2020, Elsevier

Therefore, this review systematically summarizes the rapidly evolving materials of ECNFAs, with a specific focus on the relationship between the structure and properties of ECNFAs. As shown in Fig. 1, the paper begins by systematically introducing the fabrication methods of ECNFAs and the regulatory methods for corresponding structures and then presents a comprehensive summary of the optimization methodology from nano-building blocks to structure design of ECNFAs, aiming to fabricate ECNFAs with exceptional compression resilience, outstanding flexibility, and superior stretchability. Next, remarkable potential applications in fields such as thermal regulation, environmental protection, and biomedicine are elaborately illustrated. Lastly, the current challenges and opportunities are also discussed.

## Electrospinning Ceramic Nanofibers

Electrospinning is a widely adopted method for the production of CNFs. During the electrospinning process, a pendant droplet is first extruded from the spinning solution by a syringe pump and then deforms into a Taylor cone under the influence of electrostatic repulsion and the Coulomb force generated by a high-voltage electric field. The Taylor cone subsequently elongates to produce a charged jet that flies in the high-voltage electric field. As the charged jet travels, it experiences force imbalance accompanied by solvent evaporation, generating bending instability that leads to the elongation and thinning of the jet. The process results in finer-diameter fibers that solidify more rapidly, and finally, the solidified fibers are deposited on the collector [31]. For electrospinning, the material composition plays a crucial role in determining different processing routes. For oxide CNFs (encompassing both metallic [32] and non-metallic oxides [33]), the conventional approach combines electrospinning with sol–gel processing [34, 35], which involves (i) preparing a spinnable sol containing metal alkoxides or inorganic salts, (ii) electrospinning the sol into precursor nanofibers, and (iii) calcining the nanofibers at 500–1200 °C to achieve crystallization and phase transformation into oxide ceramics. Non-oxide CNFs (e.g., carbides [36], nitrides [37], borides [38]) necessitate a precursor-derived approach, wherein polymer precursors with target elemental compositions (e.g., polycarbosilane for SiC fibers) are electrospun into nanofibrous structures and the nanofibers undergo pre-oxidation at 200–400 °C in the air to form oxidative cross-linking networks, and finally, the nanofibers are ceramicized at 1200–1800 °C under an inert atmosphere.

### Sol–gel Method

The sol–gel method involves the hydrolysis of metal inorganic salts or alcoholysis of metal alkoxides under controlled conditions, accompanied by condensation reactions to form a linear sol with a certain viscosity. These chemical reaction processes can be expressed by the following reaction equation [39]: 

Hydrolysis:

1 $${\mathrm{M}} - {\mathrm{OR}} + {\mathrm{H}}_{2} {\mathrm{O}} \to {\mathrm{M}} - {\mathrm{OH}} + {\mathrm{ROH}}$$

Condensation:

2 $${\mathrm{M}} - {\mathrm{OH}} + {\mathrm{HO}} - {\mathrm{M}} \to {\mathrm{M}} - {\mathrm{O}} - {\mathrm{M}} + {\mathrm{H}}_{2} {\mathrm{O}}$$

3 $${\mathrm{M}} - {\mathrm{OR}} + {\mathrm{HO}} - {\mathrm{M}} \to {\mathrm{M}} - {\mathrm{O}} - {\mathrm{M}} + {\mathrm{ROH}}$$

**Fig. 2:**
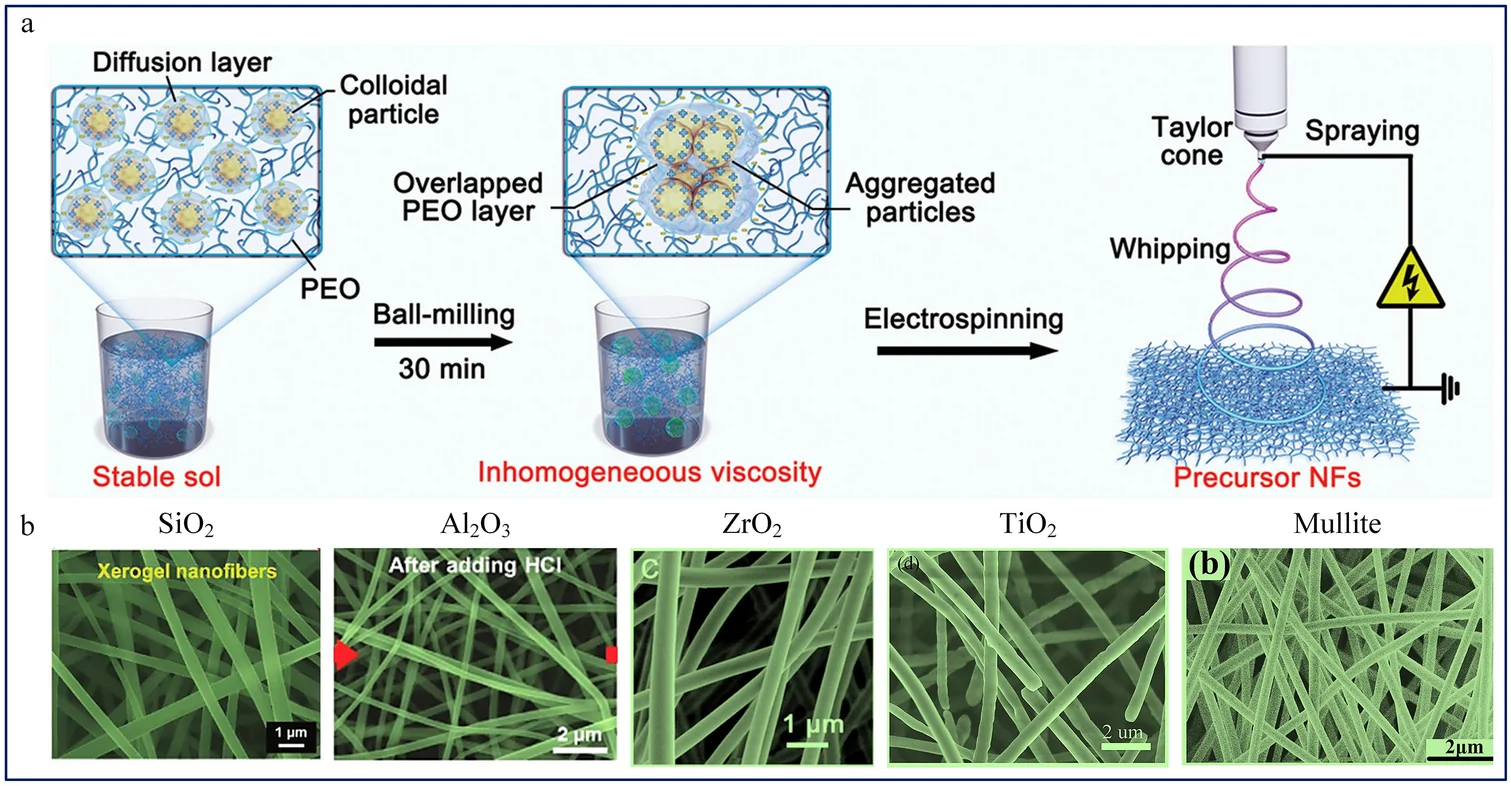
**a** Schematic representation of the sol–gel methods employed for electrospinning oxide-ceramic nanofibers. Adapted with permission from Ref. [91]. Copyright 2021, Wiley–VCH. **b** Scanning electron microscope (SEM) images showcasing various electrospun oxide-ceramic nanofibers: SiO_2_. Adapted with permission from Ref. [93]. Copyright 2023, Wiley–VCH. Al_2_O_3_. Adapted with permission from Ref. [95]. Copyright 2024, Wiley–VCH. ZrO_2_. Adapted with permission from Ref. [285]. Copyright 2021, Royal Society of Chemistry. TiO_2_. Adapted with permission from Ref. [286]. Copyright 2019, Elsevier. Mullite. Adapted with permission from Ref. [62]. Copyright 2019, Elsevier

Continuous hydrolysis and condensation lead to progressive chain elongation within the sol matrix, accompanied by a measurable increase in viscosity. As shown in Fig. 2a, upon achieving spinnable viscosity thresholds (typically 0.01–10 Pa s) [40], the sol undergoes electrospinning to generate continuous precursor jets and subsequent solvent evaporation during jet elongation promotes further condensation, ultimately yielding gel fibers and converting into the required CNFs after tailoring thermal treatment. Thus, the CNFs’ morphology and performance are mainly controlled by the hydrolysis-condensation kinetics, including precursor stoichiometry, solvent selection based on dielectric properties and volatility, and pH modulation to control reaction rates. Furthermore, the spinning aids and electrospinning operational parameters also influence the CNFs' structure. Through rational integration of sol–gel chemistry with parameter optimization protocols, a variety of oxide CNFs have been successfully synthesized (Fig. 2b), encompassing singular oxides (SiO_2_ [41, 42], ZrO_2_ [43, 44], TiO_2_ [45, 46], Al_2_O_3_ [47], mullite [48]) and composite formulations (SiO_2_/ZrO_2_ [49] and Al_2_O_3_/ZrO_2_ [50]). These ECNFs combine intrinsic ceramic advantages (thermal resilience, chemical inertness) with nano-structural benefits inherent to electrospinning (high surface-to-volume ratios, structural continuity, tunable porosity), making them promising candidates for advanced applications in catalysis, energy storage, and high-temperature filtration systems.

### Precursor-Derived Method

**Fig. 3:**
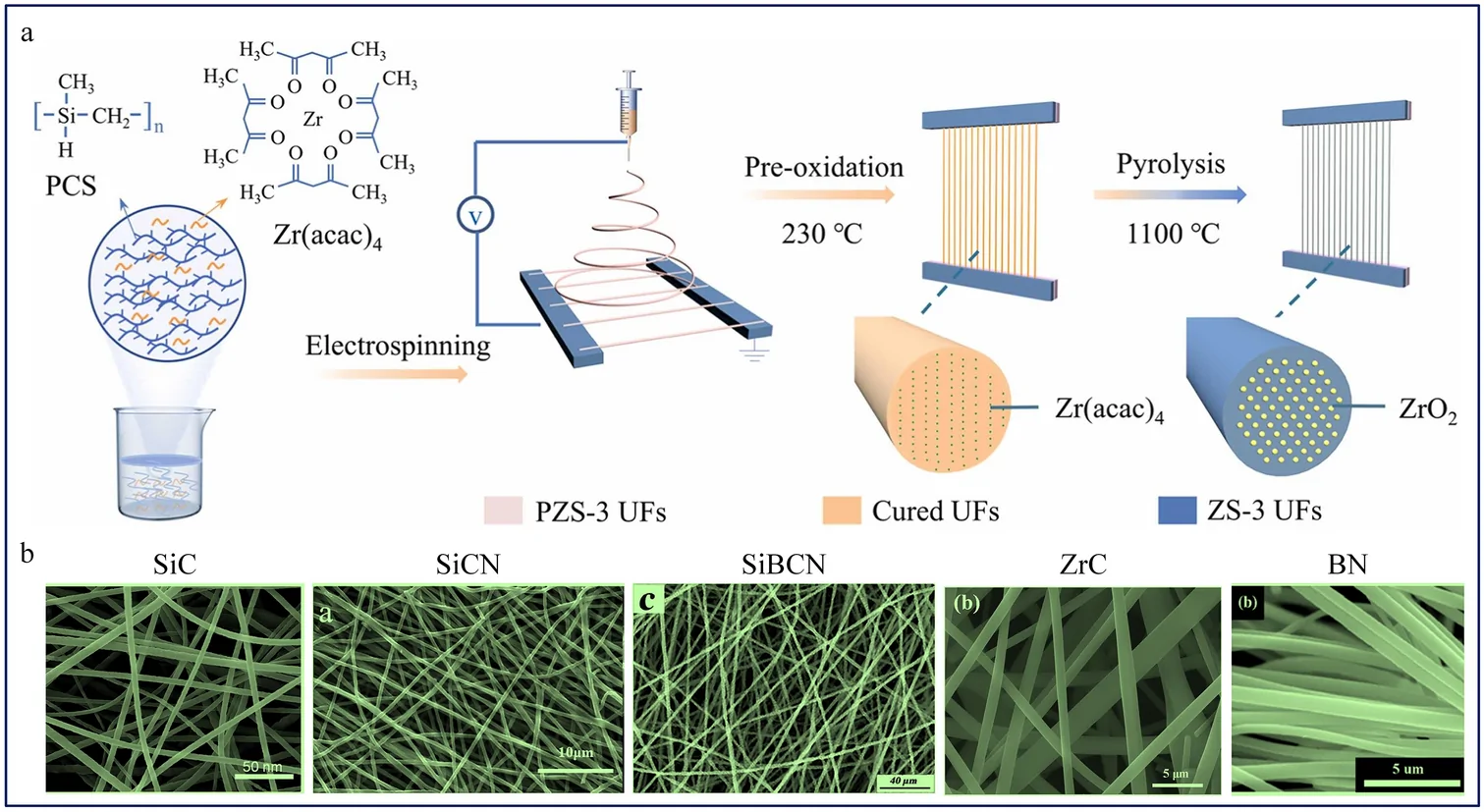
**a** Schematic diagram of the precursor-derived method for electrospinning non-oxide ceramic nanofibers. Adapted with permission from Ref. [287]. Copyright 2024, Elsevier. **b** SEM images showcasing various electrospun non-oxide ceramic nanofibers: SiC. Adapted with permission from Ref. [53]. Copyright 2023, American Chemical Society. SiCN. Adapted with permission from Ref. [288]. Copyright 2020, Elsevier. SiBCN. Adapted with permission from Ref. [56]. Copyright 2021, Elsevier. ZrC. Adapted with permission from Ref. [289]. Copyright 2022, Elsevier. BN. Adapted with permission from Ref. [58]. Copyright 2010, Spring Nature

While oxide CNFs demonstrate promising applications across multiple domains, their utilization in ultrahigh-temperature environments (> 1000 °C) remains constrained by mechanical degradation arising from crystallization-induced pulverization. Compared to the oxide CNFs, non-oxide ones exhibit superior high-temperature stability, enhanced mechanical strength/modulus, and exceptional corrosion resistance. Take SiC as an example, the precursor-derived synthesis methodology was pioneered by Yajima et al. [51] in 1975, who synthesized polycarbosilane (PCS) via high-pressure pyrolysis, subsequently processed through melt-spinning, thermo-oxidative stabilization, and calcination to obtain SiC fibers with micron-scale diameters. To decrease the diameter to nanoscale, a significant advancement occurred in 2009 when Eick et al. [52] integrated electrospinning with ultraviolet-assisted curing of polystyrene/PCS composite precursors, synthesizing the SiC nanofibers via hybrid electrospinning-precursor-derived method. As illustrated in Fig. 3a, the diverse non-oxide ceramic nanofibers have been successfully prepared according to the method, including SiC [53], SiCN [54, 55], SiBCN [56, 57], boron nitride (BN) [58], and ZrC [59, 60] (Fig. 3b). The unique thermal stability and semiconductor properties of non-oxide ceramic nanofibers make them promising candidates for aerospace thermal protection systems, ultrahigh-temperature insulation composite materials, and radar-absorbing structural materials.

## Construction of ECNFAs

ECNFs have emerged as promising building blocks for elastic ceramic aerogels due to their exceptional aspect ratios, intrinsic flexibility, and ease of scalable synthesis from various materials. While exhibiting exceptional potential, the primary limitation hindering the broader application of the ECNFs lies in their inherent tendency to form directional layered structures during fabrication, a characteristic that restricts progress in enhancing both dimensional density and void content within deposited fibrous matrices produced through electrospinning. Alternatively, 3D structured ceramic nanofiber-based aerogels possessing exceptional porosity and superior elastic deformation could offer a viable pathway to address these limitations; however, fabricating such aerogels remains substantially challenging within current production methodologies. In recent research, the method of ice-template-induced 3D self-assembly of ECNFs has been used to fabricate various ceramic nanofibrous aerogels, such as SiO_2_ NFAs [6], TiO_2_ NFAs [61], and mullite NFAs [62]. These aerogels exhibit similar compressive response characteristics through the deformation mechanism of hierarchical cellular structures. Regrettably, the point-to-point overlap structure of nanofibers in cellular walls exhibits insufficient bonding integrity to withstand intense mechanical loads during deformation processes. Multi-mode bonding forms in suitable pore structures, such as line-to-face and face-to-face bonding, can be constructed to enhance the strength of the ECNFAs by foaming and layer-by-layer stacking methods [63]. Despite the exceptional mechanical compressive properties of elastic ECNFAs, the constrained kinematic freedom of nanofibrous walls in motion fundamentally restricts the tensile deformation of ceramic aerogels. To solve the limitations, some works have mainly concentrated on the construction of curly nanofibers in aerogels by direct electrospinning techniques, thereby achieving the tensile deformation of ECNFAs. In all, the chapter systematically discusses the mainstream fabrication methods, diverse microstructure characteristics, and mechanical improvement strategies for ECNFAs.

### Fabrication and Structure

The morphological architectures of aerogels exhibit structurally versatile pore morphologies (e.g., lamellar, honeycomb, and disordered macropores), which are decided by the forming method. In the past few decades, significant advancements have been made in developing synthesis techniques for ECNFAs, such as freeze-drying, layer-by-layer stacking, foaming, and direct spinning shaping. Here, we will systematically analyze the critical relationship between fabrication strategies and the resultant pore topology, with particular emphasis on structural modulation through process optimization.

#### Freeze-drying

**Fig. 4:**
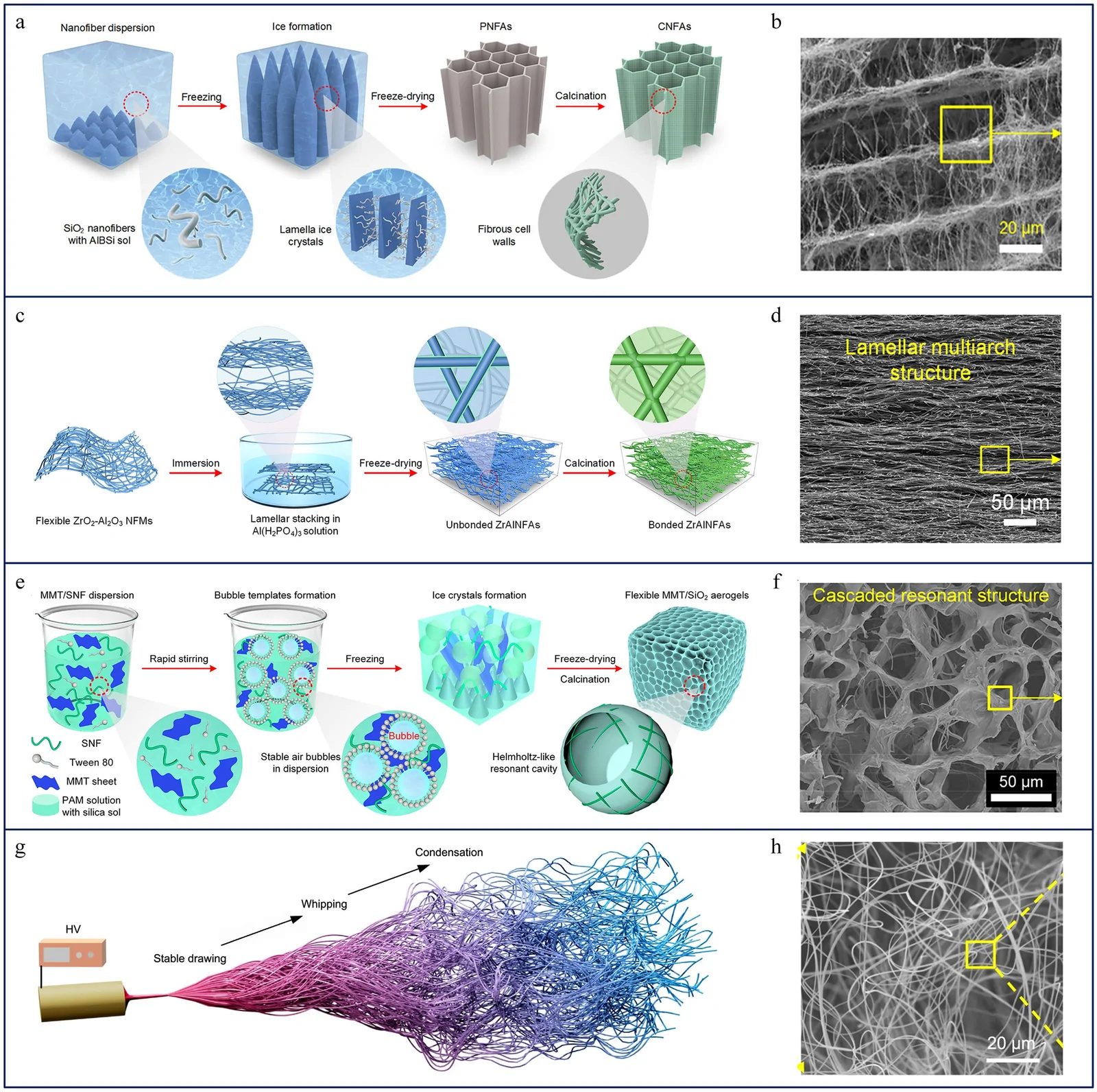
**a** Schematic illustration of the freeze-drying process and** b** the corresponding SEM image. Adapted with permission from Ref. [65]. Copyright 2018, American Association for the Advancement of Science. **c** Schematic representation of layer-by-layer stacking method and **d** the corresponding SEM image. Adapted with permission from Ref. [68]. Copyright 2020, American Chemical Society. **e** Schematic illustration of the foaming process and** f** the corresponding SEM image. Adapted with permission from Ref. [73]. Copyright 2022, American Chemical Society. **g** Schematic representation of the direct spinning and **h** the corresponding SEM image. Adapted with permission from Ref. [79]. Copyright 2022, Springer Nature

The freeze-drying method, utilizing ECNFs (length range 10–200 µm) as primary structural units, has emerged as a reliable method for fabricating elastic ECNFAs with programmable cellular structures (Fig. 4a, b). During the process, the ECNFs are extruded by the advancing ice crystal front in a sub-zero environment, ultimately forming a porous structure composed of interwoven nanofibrous cell walls after ice sublimation. Thus, the structural integrity of elastic ECNFAs critically depends on the synergistic effects of multiple variables, including the bulk ECNFs characteristics, nanofiber dispersion state in suspension, ice crystal configuration, and drying method. Among them, the cell walls formed by the ECNFs with excessive length (> 1 mm) may cause gravitational collapse of the ECNFAs structure, whereas those constructed from ultra-short ECNFs (< 10 µm) lack elastic deformation due to insufficient fiber entanglement [6, 64]. The discrete nanofiber dispersion dynamics in the suspension also govern the microstructures of the ECNFAs. The cationic polymer-assisted dispersing nanofibers within aqueous systems facilitate the formation of ECNFAs with uniform cellular architecture and isotropic pore distribution [6, 65]. Furthermore, external electric field-assisted nanofiber dispersion induces directional alignment of nanofibers along the field, thereby generating aerogels with long-range ordered cellular architectures [66]. During the freezing process of nanofiber suspension, the kinetics of ice crystal growth, governed by cooling rates, precisely regulate aerogels’ pore morphology: (i) slow cooling rate enables heterogeneous nucleation that produces a lamellar structure; (ii) moderate cooling rates facilitate secondary ice bridging to form a hierarchical honeycomb network; (iii) rapid cooling kinetically traps nanofibers in metastable lapped configurations with suppressed structural ordering [67]. Moreover, controlling the direction of heat transfer can achieve the same effect, such as replacing high-conductivity copper molds with polytetrafluoroethylene, which enforces vertical ice growth to refine lamellar architectures. Lastly, the drying method, for example, atmospheric pressure (APD), supercritical (SD), or freeze-drying (FD), can always affect the size of the ECNFAs pore structure, as shown in Table 1. All in all, the complex and diverse ECNFAs’ cellular structure can be fabricated by controlling nanofiber engineering, dispersion thermodynamics, crystallization kinetics, and drying form.

**Table 1:** Comparison of different drying methods

Method	Mechanism	Advantages	Limitations
APD	Solvent evaporation under atmospheric conditions	Low cost, simplicity	Requires ultralow surface tension dispersants
SD	CO₂ critical point transition	Minimal shrinkage, high porosity	High safety risks, scalability challenges
FD	Sublimation under vacuum	Structural versatility, no collapse	Energy-intensive

#### Layer-by-Layer Stacking

The layer-by-layer (LBL) stacking technique represents an advanced progression of nanofibrous freeze-drying methodologies, which utilize electrospun ceramic nanofiber membranes (ECNMs) as core structural components instead of discrete ECNFs. As demonstrated in Fig. 4c, rectangular ECNMs are first immersed in an aqueous ceramic sol solution and then subjected to freeze-drying to synthesize ECNFAs with hierarchical multi-arch architecture. Specifically, within the architecture, the lamellar interfacial bonding structure maintains the structural integrity of the aerogels (Fig. 4d). Compared to nanofibrous interwoven cellular structure, the multi-arch architecture can endow the ECNFAs with superior elasticity (> 90% strain recovery) and enhanced compressive strength (0.5–3 MPa) [68]. Furthermore, their pore structure can also be precisely tunable through the ECNM stacking density. For instance, the ultrasonically assisted dispersion applied during the ECNMs’ layer-stacking process facilitates the loosening of dense nanofibrous membrane structures and thereby lowers the interlayer density of the ECNFAs, which exhibit a cell wall thickness of 50–200 nm and interlayer spacing of 5–20 µm [69].

#### Foaming

The foaming method provides a versatile and scalable approach for fabricating ECNFAs by transforming two-dimensional nanofiber membranes into three-dimensional porous networks through in situ gas templating, wherein gas generation, achieved via chemical reactions (e.g., catalytic decomposition of H_2_O_2_) or inert gas injection, enables the rapid formation (within < 5 min) of interconnected pore structures, demonstrating exceptional scalability [70, 71]. Ko et al. [72] fabricated nanofibrous aerogels by immersing densely packed ECNMs in a solution containing H_2_O_2_. During the catalytically induced decomposition of hydrogen peroxide, oxygen microbubbles formed within the fibrous matrix. These gas pockets physically separated adjacent fiber clusters, thereby transforming the original flat ECNMs with overlapping fibers into a hierarchically porous layered structure, ultimately yielding a three-dimensional aerogel scaffold composed of interconnected ECNMs. Thus, the pore structure of aerogels largely depends on the foaming kinetics, including the type and concentration of the foaming agent. For example, a small amount of NaBH_4_ with a molar amount between 0.15 and 0.25 mol enables the formation of aerogels with a uniform multi-layered structure and relatively higher porosity (50%-70%), while a quantity exceeding 0.25 mol leads to pore collapse, causing volumetric shrinkage due to excessive gas evolution [71]. Although the direct foaming method enables facile fabrication of compositionally varied ECNFAs, it inherently generates architecturally single and uncontrolled pore distribution. Recent studies have proposed an innovative strategy to enrich the pore structure by combining foaming and rapid freezing techniques. The dual-templating approach, utilizing gas bubbles and solvent ice crystals (Fig. 4e), generates hierarchical architecture with nested macropores (10–50 μm) and minor pores (1–2 μm), increasing structural diversity [73]. Concretely, Zong et al. introduced Tween 80 into a mixed dispersion of SiO_2_ nanofibers, montmorillonite (MMT) nanosheets, and SiO_2_ sol to generate a stabilized foam with microbubbles, which act as macropore templates. Meanwhile, the solvent forms ice crystals during freezing, compelling SiO_2_ NFs and MMT into interlamellar walls with minor pores (1–2 μm). The resulting ECNFAs featuring a cascaded resonant cavity structure with macropore cavities and minor-pore walls (Fig. 4f) were successfully obtained after the drying process.

#### Direct Spinning

Integrating sol–gel chemistry with electrospinning presents inherent challenges in achieving self-supporting 3D architectures, primarily attributed to residual solvent retention within freshly deposited gel nanofibers, resulting in nanofibers with low strength and strong adhesion between them. The phenomenon combined with the effect of gravitational settling during vertical stacking promotes collapse into 2D films rather than porous 3D networks. While increasing solvent volatilization rates during jet flight can enhance pre-deposition solidification [74], such strategies are constrained by strong electrostatic attraction from the collector that induces fiber alignment and compaction.

Recent advances address these limitations through synergistic physicochemical interventions. For instance, electrostatic repulsion forces induced via static electric field manipulation (e.g., electrostatic induction and polarization) [74–76], enable preliminary 3D layering but suffer from weak repulsion in the thickness direction, resulting in anisotropic stacking. To overcome the limitation, counter-field perturbation systems—such as coaxial aerodynamic swirling [77] and conjugate high-voltage electrodes [78]—have been employed to disrupt electrostatic alignment, amplify jet whipping instabilities, and promote isotropic fiber entanglement. However, the complexity of these systems hinders scalable ECNFAs’ fabrication. A simpler alternative involves inducing out-of-plane curling of deposited gel nanofibers to create vertically oriented spring-like units that mechanically resist compaction and enhance pore accessibility. The key to the successful implementation of the method is to control rapid jet curing kinetics to preserve helical configurations, necessitating simultaneous control of in-flight coiling dynamics and post-deposition geometry retention. Cheng et al. [79] found that elevated sol protonation levels critically regulate curing dynamics: high protonation states intensify hydrolysis-condensation site density and enhance solution conductivity, synergistically accelerating solvent evaporation-driven condensation while amplifying jet whipping instability (Fig. 4g). The dual mechanism kinetically traps helical nanofiber configurations, which are organized into interconnected 3D aerogels (Fig. 4h) via vertical collector motion. Their work established the first quantitative framework correlating jet curing kinetics to ECNFAs’ microstructure, formalized as the "3D reaction spinning" methodology [79, 80]. The principle of self-supporting molding of curled nanofibers of ECNFAs can also be further extended through solvent-mediated (such as concentration and solvent vapor pressure) curing control [81]. Collectively, these advances demonstrate that ECNFAs’ architecture can be rationally engineered through sol protonation-driven reaction kinetics and solvent evaporation thermodynamics, establishing self-support of curled fibers as an effective method for direct preparation of ECNFAs by electrospinning.

### Optimization of Mechanical Properties

**Fig. 5:**
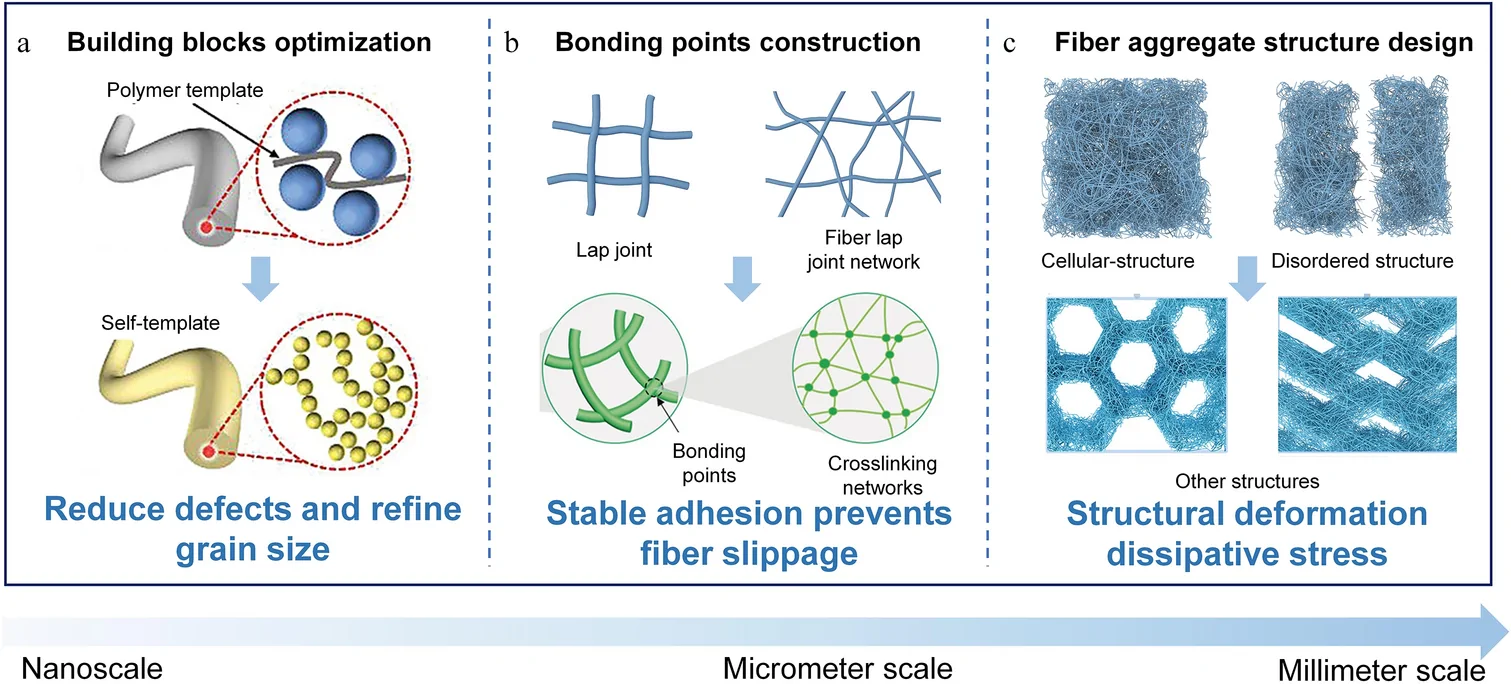
Schematic illustration of optimization strategies for ECNFAs across various scales. **a** Illustration of the optimization of building blocks optimization. Adapted with permission from Ref. [93]. Copyright 2023, Wiley–VCH. **b** Mechanism of enhancement through bonding points constructions. Adapted with permission from Ref. [275]. Copyright 2020, Wiley–VCH. **c** Optimization mechanism related to fiber aggregated structure design. Adapted with permission from Ref. [104]. Copyright 2024, Wiley–VCH

Despite advances in tailoring ECNFAs with customizable structures and functionalities, their practical deployment remains constrained by insufficient mechanical properties induced by the inherent brittleness of ceramic components, which precipitates catastrophic strength degradation and structural collapse under thermomechanical stresses, including cyclic loading and thermal shock [14, 82, 83]. To address these limitations, recent advances have established three hierarchical reinforcement strategies: (i) enhancing intrinsic building block toughness and strength (Fig. 5a) through phase engineering, doping strategies, and advanced fabrication techniques of ECNFs; (ii) microscale interfacial bonding design (Fig. 5b), leveraging a large amount of covalent/non-covalent junctions to strengthen fiber junctions; and (iii) macroscale architectural customization (Fig. 5c), controlling nanofiber aggregate structures (e.g., gradient or hierarchical honeycombs) to redirect stress distributions. These strategies cover multiple length scales from molecular to macrostructure, providing a comprehensive framework for optimizing the mechanical properties of ECNFAs [84].

#### Building Blocks

ECNFs, as core load-bearing units, fundamentally determine the mechanical and structural stability of ECNFAs [5]. Enhancing the mechanical performance of ECNFs necessitates precise engineering of their internal microstructure, including the morphology, size, arrangement, and combination of crystal and amorphous regions, the content and distribution of cracks, defects, and heterogeneous interfaces. Therefore, three synergistic strategies have been presented for enhancing ECNFs: i) phase engineering mainly involving crystallographic phase modulation; (ii) doping the second elements into the nanofibers to induce crack deflection and stress redistribution; (iii) advanced processing to control grain refinement and alignment. These strategies effectively optimize the internal microstructure of ECNFs, enabling the fabrication of ECNFAs with unprecedented mechanical robustness.

**Fig. 6:**
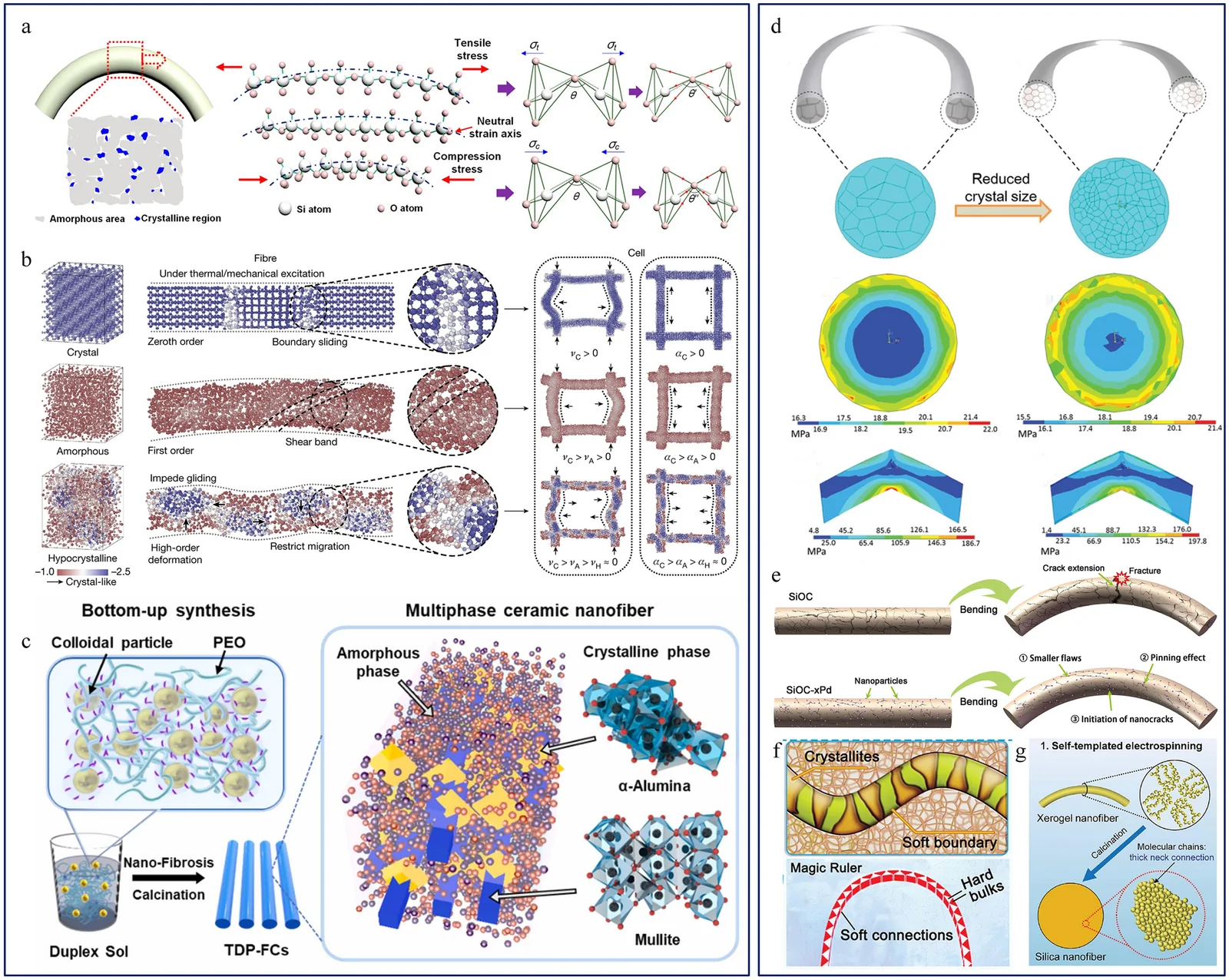
**a** Enhancement mechanism of SnO_2_ nanocrystals embedded within an amorphous SiO_2_ matrix. Adapted with permission from Ref. [85]. Copyright 2017, American Chemical Society. **b** Deformation modes of hypocrystalline. Adapted with permission from Ref. [77], Copyright 2022, Springer Nature. **c** Schematic diagram of multi-crystalline phases. Adapted with permission from Ref. [86]. Copyright 2022, Elsevier. **d** Enhancement mechanism of ionic doping optimization. Adapted with permission from Ref. [88]. Copyright 2020, Wiley–VCH. **e** Optimization mechanism related to nanoparticle doping. Adapted with permission from Ref. [90]. Copyright 2018, American Chemical Society. **f** Optimization mechanism involving ball-milling spinning sol and the curved stretching precursor fiber. Adapted with permission from Ref. [91]. Copyright 2021, Wiley–VCH. **g** Optimization mechanism of self-template strategies. Adapted with permission from Ref. [93]. Copyright 2023, Wiley–VCH

*Phase engineering strategies *Phase engineering leverages the synergistic integration of amorphous matrices and nanocrystalline domains to create heterogeneous grain boundaries that deflect crack propagation and dissipate strain energy, effectively overcoming the intrinsic brittleness of ECNFs. A typical example is SnO_2_-doped SiO_2_ nanofibers, where SnO_2_ nanocrystals (5–20 nm) act as rigid "bricks" embedded within an amorphous SiO_2_ "mortar" matrix (Fig. 6a). The brick-and-mortar architecture can introduce many heterogeneous interfaces that impede crack advancement through crack tip bifurcation and interfacial debonding, achieving a tensile strength of 4.15 MPa, a 320% enhancement over monolithic SiO_2_ nanofibers [85]. Similarly, Guo et al. [77] engineered hypocrystalline zirconium silicate fibers with nanocrystalline domains (< 10 nm) pinned by an amorphous matrix, where the amorphous phase restricts nanocrystal sliding while nanocrystals inhibit amorphous phase migration at high temperatures (Fig. 6b). The dual inhibition mechanism achieves unprecedented mechanical properties, including 7.9 GPa tensile strength and 6.7% strain tolerance. Furthermore, the mutual inhibition effect of multi-crystalline phases and the synergetic effect of the crystalline-amorphous phase can also improve the mechanical properties of nanofibers. For example, by incorporating α-Al_2_O_3_/mullite double grain interface into a multiphase system (Fig. 6c), a three-phase mode can be constructed, wherein the geometric mismatch at grain boundaries generates dislocations that suppress grain coalescence, while the amorphous phase buffers stress during deformation. The design of the multiphase structure enables the aerogels to have 80% compressive strain recovery and retain structural integrity after a 30-min exposure to 1600 °C, outperforming state-of-the-art ceramic aerogels [86].

*Doping strategies* Doping serves as a pivotal strategy for refining the mechanical properties of ECNFs by reducing grain size and inducing lattice distortion, which includes ionic, atomic, and nanoparticle doping [87]. In ionic doping, a prominent example is that Y^3+^ doping in TiO_2_ nanofibers enables the nanofibrous membranes to withstand over 20,000 bending cycles [88]. The enhancement mechanism (Fig. 6d) mainly originates from grain boundary segregation suppressing atomic diffusion, refining grain size, and intensifying grain distribution to disperse crack propagation. Meanwhile, grain boundary sliding, according to the Hall–Petch effect, enhances strength and superplasticity, enabling polycrystalline fibers with ultrafine grains to withstand more severe deformations, while dispersed Y oxides act as solid lubricants and intergranular bridges (Y–OH bonds), dissipating external forces during deformation. Unlike ion doping, carbon atomic doping can form a certain number of C/TiO_2_ heterointerfaces within nanofibers, and simultaneously the introduction of C atoms can also disrupt the regular lattice of TiO_2_, inducing lattice distortion. The combined effect of heterointerfaces and lattice distortion effectively redistributes stress, increasing the tensile strength of TiO_2_ nanofibers from 0.5 to 2.5 MPa [89]. Additionally, nanoparticle doping can enhance ECNFs by reducing defects and introducing microcracks to achieve effective stress and energy dissipation. For example, as demonstrated in Fig. 6e, when SiOC nanofibers are doped with Pd nanoparticles, the nanoparticles can repair the defects within the nanofibers and dissipate the stress by generating cracks caused by the difference in expansion coefficients, thus avoiding stress concentration and ultimately achieving the effect of strengthening SiOC nanofibers [90].

*Advanced processing strategies* Advanced processing techniques have emerged as innovative approaches to enhance the mechanical performance of ECNFs by minimizing structural flaws and optimizing nanofiber alignment. Ball-milling spinning sol and stretching precursor nanofibers have been proven to be an effective strengthening strategy by reducing defects, refining grain size, and aligning grains in nanofibers (Fig. 6f), and ultimately achieving a 40% improvement in flexibility and tensile strength [91]. However, the above method still relies on polymer templates to ensure spinning continuity and enhance the mechanical stability of ECNFs, the removal of which often introduces porosity and compromises structural integrity [92]. The self-templating method was developed to eliminate the negative impact brought by polymer templates, which involves concentrating on the ceramic precursor sol to form highly active, long-chain inorganic molecules resembling polymers (Fig. 6g). The enhanced mechanism of nanofibers by the self-templating method mainly refers to reducing intergranular voids and promoting crystalline phase homogeneity [93]. For instance, Al_2_O_3_ nanofibers, fabricated by the self-templating method, demonstrated a tensile strength of 1.02 GPa, which was 4.6 times higher than that of the nanofibers templated by polymers [94]. Spectacularly, the ECNFAs constructed by the Al_2_O_3_ nanofibers manifested remarkable high-temperature flexibility, enduring thousands of bending cycles at 1700 °C without fracture [95]. Moreover, by precisely controlling the calcination parameters, such as maintaining heating rates below 5 °C min^−1^, the mechanical properties of the materials can likely be optimized.

In conclusion, these advances highlight the transformative potential of defect-minimized processing routes in fabricating ultra-strong, thermally resilient ECNFs for extreme-condition applications. As summarized in Table 2, the synergy of phase engineering, doping, and advanced processing enables ECNFAs to overcome traditional ceramic brittleness, achieving mechanical robustness (single nanofiber’s tensile stress ˃ 7.9 GPa) and high-temperature stability (˃ 1700 °C). These strategies collectively advance applications in extreme environments, such as aerospace thermal barriers and industrial catalysis.

**Table 2:** Comparison of properties of ceramic nanofibers prepared by electrospinning

Fiber type	Enhancement strategy	Fiber diameter (nm)	Young's modulus (MPa)	Tensile stress (MPa)	Bending rigidity (mN)	Other mechanical properties	Refs
SiO_2_	/	290	320	7.1	13.11	/	[256]
	Self-template	200	/	Single fiber: 1.41 GPa	/	Toughness: 34.29 MJ m^−3^	[93]
	SnO_2_-doped	97 ~ 339	/	2.48 ~ 4.15	22 ~ 36	Toughness: 0.056 ~ 0.016 MJ m^−3^	[85]
ZrO_2_/SiO_2_	SiO_2_-doped	500 ~ 800	/	1.68		Toughness: 0.32 MJ m^−3^	[257]
zircon	hypocrystalline	700	Single fiber: 173.5 GPa	Single fiber: 7.9 GPa		Strain 6.7%	[77]
ZrO_2_	Y^3+^-doped	79 ± 7	133 ~ 362	/	/	Hardness 0.86 ~ 1.67 MPa	[258]
	Y^3+^-doped	270	/	4.82	26	/	[92]
	Y^3+^-doped	282	539	5.04	24	Toughness 0.026 MJ m^−3^	[259]
	Y-SiO_2_-doped	~ 1000	/	5.9 ± 0.8	/	/	[49]
	Al_2_O_3_ coating	200	/	/		Bending angle of 180°	[260]
	SiO_2_-doped	/	/	1.83	0.2 cN mm^−1^	/	[261]
Al_2_O_3_/ZrO_2_/Y_2_O_3_	Al-Zr-Y-doped	498 ± 100	/	/	/	Bending angle 0 ~ 360°	[262]
Al_2_O_3_	Self-template	~ 500	/	Single fiber 1.02 GPa	/	/	[94]
	/	200 ~ 250	/	2..98	/	Elongation at break 1.5%	[263]
	/	115 ~ 128	127/134	1.78/1.51	/	/	[264]
YAG	Al_2_O_3_-doped	300 ~ 350	/	3.52 ± 0.31	/	/	[265]
TiO_2_	Y^3+^-doped and g-C_3_N_4_ coating	240	/	1.28	27	/	[266]
	C-TiO_2_-doped	205	104	0.516 ~ 0.573	14 ~ 41	Folding angle 180°	[135]
	CeO_2_-doped	350	/	1.38	/	Elongation at break 1.3%	[267]
	Soft Zr-doped	304 ~ 371	120.7	1.32	26	Elongation at break 2%	[268]
	Ball-milling, curved-drafting	400 ~ 700	30.2	0.62	22	Strain 2.05%, Elastic modulus 20.8 GPa	[91]
	C-doped	180	/	2.5	10	Folding angle 180°, Bending angle 120° to 45°	[89]
HfC/SiC	H_f_C-doped	260 ~ 980	/	11	/	/	[269]
SiC	Al-doped	2830 ~ 3840	/	1.07 ± 0.34	/	/	[270]
	Hot drawing	~ 1000	/	243 ± 17.5	/	/	[271]
SiZrOC	Si-ZrO-C doped	405 ~ 450	/	0.812 ± 0.086	0.11 ± 0.01	/	[272]
	Si-ZrO-C doped	588.2	/	0.821	/	/	[273]
SiOC	Pd-doped	550	Elastic modulus 466 MPa	33.2	0.022 cN cm^−1^	Bending angle: 55° and Bending modulus: ~ 2.23 kPa	[90]

#### Bonding Point

In ECNFAs, the bonding points between nanofibers critically govern elastic deformation behavior. Simple overlapping configurations often result in fiber slippage under mechanical stress, ultimately leading to incomplete elastic recovery (< 60%) and compromised structural integrity [96]. To address the limitations, different bonding strategies have been developed to enhance the structural robustness of ECNFAs, such as point-to-point contact, line-to-face interaction, and face-to-face bonding.

*Point-to-point bonding *As the earliest and most extensively studied interfacial strategy for ECNFAs, the point-to-point bonding method employs binders such as polymers (polybenzoxazine (PBZ) [6], blocked isocyanate prepolymer (BIP) [97]) or inorganic sols (Si-sol [65], AlBSi sol [53], and soluble phosphate Al(H_2_PO_4_)_3_ [68]) to establish chemical bonds between nanofibers. For instance, AlBSi sol constructs numerous bonding points among SiO_2_ nanofibers through Si–O–Si cross-linking reaction, and the resultant SiO_2_ nanofibrous aerogels can withstand 80% compressive strain, triple the elasticity (*ε* < 5%) of conventional ceramic networks [65]. The work laid a foundation for bonding-mediated structural stabilization, and many elastic oxide ceramic nanofibrous aerogels have been developed, such as TiO_2_ NFAs [32], Gd_2_O_3_/Bi_2_O_3_ NFAs [98], α-alumina/mullite/SiO_2_ NFAs [86]. However, the bonding function of ceramic sols will be ineffective on the surface of non-oxide ceramic nanofibers (e.g., SiC) because of their chemical inertness. To address the defect, Zhang et al. [53] developed a covalent interfacial engineering strategy by utilizing the moderate rheological properties of the AlBSi sol at PCS cross-linking temperatures, achieving a robust bonding between the SiC nanofibers. Beyond sol-based approaches, Liu et al. [99] innovatively employed supramolecular self-assembled BN nanofibers as continuous fibrous binders for C/Al_2_O_3_ frameworks. The effect of bridging and encapsulation from BN nanofibers maintains 90% stress retention and 50% compressive strain after 10,000 cycles for the BN/C/ Al_2_O_3_ ceramic meta-aerogel, and the effect can avoid the failure of binders at complex operating conditions.

*Line-to-Face Bonding* Compared to the point-to-point bonding mode, the line-to-face bonding method in the form of 1D nanofibers and 2D nanosheets enhances the structural stability of ECNFAs [100–102]. Here, more interfacial contact points mean stronger interactions between the nanofibers and nanosheets, which effectively distribute external forces and reduce the likelihood of structural failure, thus enhancing the overall stability of ECNFAs. For example, integrating GO nanosheets into ECNFAs via directional freeze-drying and ascorbic acid reduction can achieve a 64% increase in compressive strength (17.2 kPa at 80% strain) compared to conventional point-to-point bonded aerogels and withstand 1000 compression cycles at 60% strain with minimal plastic deformation [101]. These interactions between the nanofibers and nanosheets also enable the ECNFAs’ stretchability with a tensile stress of up to 12.56 kPa and a strain of 4.8%.

*Face-to-Face Bonding* The face-to-face bonding method optimizes mechanical properties by maximizing interfacial bonding points among nanofibers, which can transform weak point-to-point connections into a robust face-to-face bonding form in ECNFAs. For instance, Zhang et al. [68] convert sparse fiber junctions into dense face-to-face contacts by engineering layer-by-layer stacking of ECNMs (Fig. 6c), which redistribute stress across interconnected planes, achieving compressive strengths (> 301 kPa at 80% strain) one to two orders of magnitude higher than conventional bonded ECNFAs. Subsequently, the group [100] further advanced the strategy by incorporating MMT nanosheets into ECNFAs preparation by layer-by-layer stacking. The line-to-face interactions between fibers and MMT further expanded stress dissipation pathways, enabling the aerogel to withstand 317 kPa compressive stress at 80% strain while exhibiting exceptional tensile (359 kPa) and bending (319 kPa) strengths-surpassing other aerogels by an order of magnitude.

#### Nanofiber Aggregate Structure

The mechanical behavior of ECNFAs is also governed by their structural architecture, which determines the mechanisms through which stress is absorbed, transmitted, and redistributed during deformation under loading conditions. By tailoring these architectures, researchers can precisely tune properties like compression elasticity, stretchability, and high mechanical strength to meet application-specific demands.

**Fig. 7:**
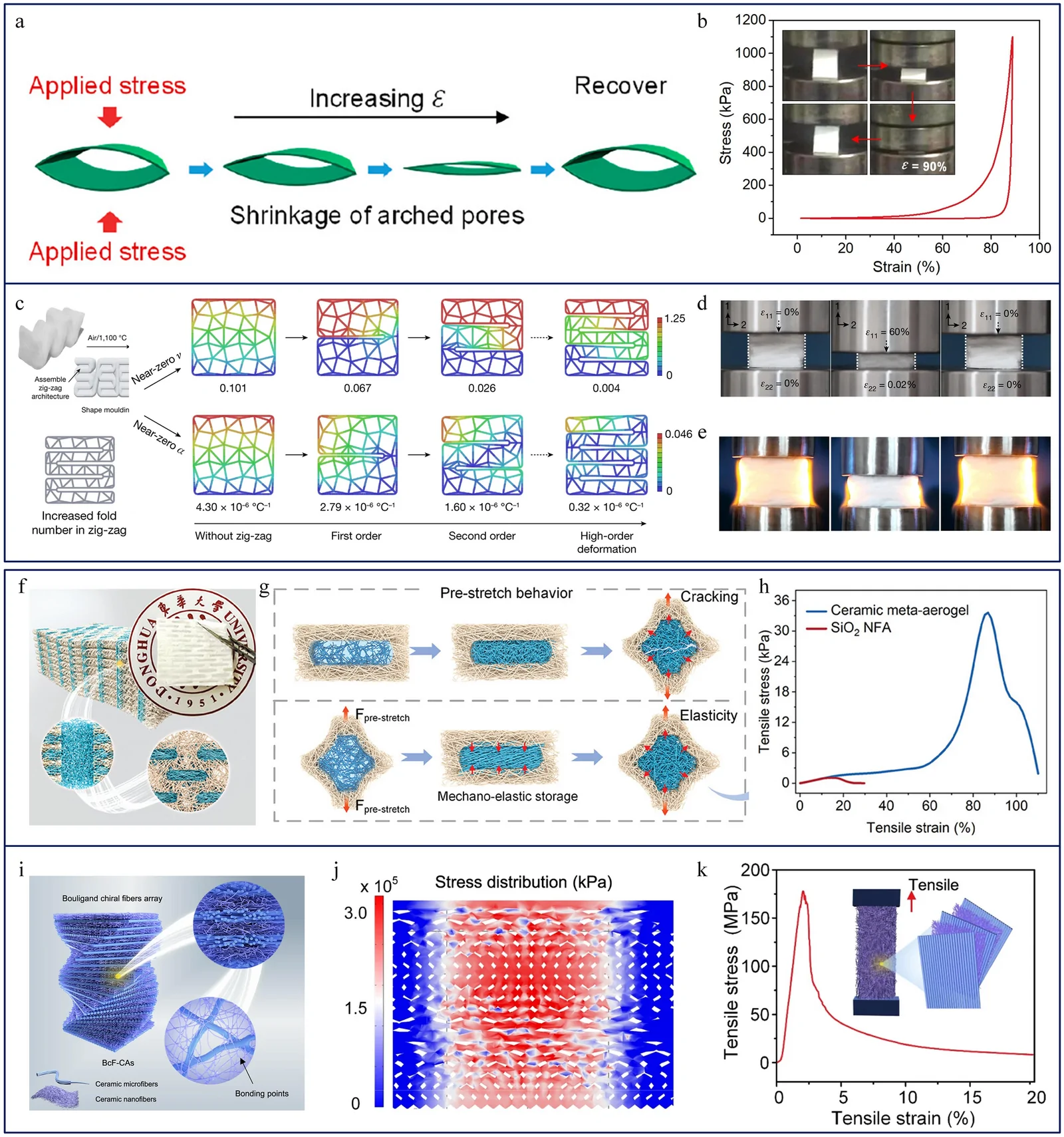
**a** Schematic representation of the deformation mechanism of the multi-arch structure under applied force and **b** the corresponding compressive stress–strain curve. Adapted with permission from Ref. [68]. Copyright 2020, American Chemical Society. **c** Illustration of the near-zero Poisson's ratio and the near-zero expansion coefficient design of zigzag architecture and **d** the near-zero Poisson's ratio and** e** the near-zero expansion coefficient in ZAGs. Adapted with permission from Ref. [77]. Copyright 2022, Springer Nature. **f** Schematic images, **g** the pre-stretch behavior and **h** the tensile stress–strain curves of ECNFAs [104]. Copyright 2024, Wiley–VCH. **i** Schematic illustration representing ECNFAs with Bouligand structures,** j** Graphical representation of in-axial tensile stress simulation, and **k** tensile stress–strain curve [105]. Copyright 2024, Springer Nature

*Structural Designs for Compressive* Cellular architecture represent one of the most effective compressive stress-dissipation designs in ECNFAs, which operate through a hierarchical multi-scale mechanism. At the macroscale, entangled nanofiber cell walls redistribute compressive loads via flipping motions, enabling strain delocalization. Mesoscopically, single nanofiber absorbs energy through bending deformations. As a typical case, the SiO_2_ nanofiber aerogels with cellular structure prepared via the freeze-drying method demonstrated the multi-scale stress-dissipation synergistic effect, achieving 70% retention of maximum stress (10.5 kPa) and Young’s modulus after 500 cycles, far surpassing the performance of conventional ceramic aerogels [65]. To enhance the compressive strength of ECNFAs, a hierarchical multi-arch architecture has been engineered to dissipate stress through the buckling deformation of arched cellular units (Fig. 7a). Concretely, the arched cell walls can transform into parallel planes during compression and maximize the load-bearing contact area, enabling face-to-face stress redistribution, achieving a compressive strength of 1100 kPa (Fig. 7b) at 90% strain of the ECNFAs. Interestingly, the aerogels with high compressive strength can withstand a load exceeding 60,000 folds their own weight while maintaining full structural recovery post-unloading, attributed to their geometry-driven elastic resilience [68]. Despite these advancements, enhanced compressive strength is generally effective only at room temperature, and ECNFAs continue to experience structural degradation when exposed to elevated temperatures. Recent research indicates that the near-zero Poisson's ratio (*ν*) could help reduce or eliminate the excessive stress induced by the longitudinal deformation and achieve a near-zero transverse strain, which may offer a design concept to optimize the structural stability of ECNFAs [77]. Consequently, the hypocrystalline zirconium silicate nanofiber ceramic aerogels with a zigzag structure (ZAGs) were designed (Fig. 7c), wherein a densely packed arrangement of layered fibers at corner points provides sufficient structural deformation stiffness, and the combination of the constraints imposed on internal fiber deformation by the outer fiber layers forms a nearly zero Poisson's ratio phenomenon. As shown in Fig. 7d, near-zero *ν* combined with the additional elasticity provided by the bending moment at the corner joints in the sawtooth structure promotes the aerogel to recover from compression with less residual strain, thereby achieving an ultimate compressive stress of 86.8 kPa and a maximum strain of 95%. At the same time, the zigzag structure design can also extend the near-zero expansion behavior of the hypocrystalline nanofibers from the local aerogel to the global ECNFAs (Fig. 7e), thereby greatly enhancing thermal stability [77].

*Structural Designs for Tensile* The optimal strategy for achieving reversible tensile deformability in the ECNFAs involves engineering internally architected spring-like fiber networks through controlled helical configurations. Cheng et al. constructed a 3D interwoven structure of curling ceramic nanofibers through the electrospinning method, which can stretch up to 100% strain and tensile recovery exceeding 40% [79]. The main factors for tensile deformation of the ECNFAs are the following: i) the highly porous entangled structure provides ample deflection space for stress dissipation; (ii) the entangled structure disperses the tension it receives at entanglement points across multiple fibers, achieving effective stress redistribution; (iii) the ECNFs possess a curved structure, which is both flexible and robust, preventing fracture before the aerogel structure fails. Similarly, Dang et al. [103] designed curled ECNFAs by incorporating two precursors with differing elastic moduli and solvent evaporation rates during the spinning process, leading to the natural formation of highly flexed structures in the resulting nanofibers. The bent nanofibers then entangled, forming an interwoven network structure within the aerogels, enabling ECNFAs to withstand 150% tensile deformation without fracture. Unfortunately, the specialized nature of the sol–gel’s electrospinning technique imposes significant constraints on the expansion of the types of curled nanofibers. The new strategy pays more attention to the collaborative design of the integral structure of ECNFAs. Zhang et al. [104] developed stretchable ECNFAs with Kirigami laminar aerogels (grid region) and nanofibrous deposited aerogels (mesh region) via a combination of paper-cut stacking and nanofiber freeze-drying techniques (Fig. 7f). The design can ingeniously convert the energy between the grid area and the mesh area, concretely, under the external force, the compressive potential energy pre-stored in the mesh aerogels is released and converted into tensile potential energy in the grid aerogels, enabling the ECNFAs to bear tensile deformation. Upon removal of the external force, the tensile potential energy released in the mesh aerogels, which comprises both the input potential energy and the converted compressive potential energy, allows the integral aerogels to rapidly return to their original state (Fig. 7g). The ECNFAs ultimately demonstrate exceptional elastic performance, achieving a maximum tensile strain exceeding 85% and withstanding 500 cycles of 50% cyclic loading without residual plastic deformation (Fig. 7h). Additionally, the high tensile strength of the ECNFAs is studied. Wang et al. [105] employed an oriented assembly technique to arrange micro-/nanofibers into helical hierarchical arrays, resembling the Bouligand chiral structure (Fig. 7i). In the configuration, a portion of the fibers undergoes tensile deformation via a stretch/slip mechanism, while the remainder rotates symmetrically away from the stress axis (Fig. 7j). Moreover, elastic modulus oscillations within the Bouligand geometry enhance the torsional effect of the cracks. Due to the stress dissipation, crack torsion in the micro-/nanoscale Bouligand arrays, and mechanical reinforcement from the micrometer fibers, the aerogels exhibited a tensile strength of 170.38 MPa along the vertical helical fiber arrays and exceptional hyperelasticity of 156.47 kPa at 80% strain (Fig. 7k) in the direction of these arrays.

In conclusion, enhancing the mechanical properties of aerogels is crucial for preserving structural integrity and optimizing performance. Fundamentally, the mechanical properties can be enhanced by reinforcing the building blocks, and appropriate enhancement strategies should be chosen accordingly. Crystalline ceramic fibers, for instance, are typically reinforced and toughened by constructing biphasic or multiphasic structures. Besides, the mechanical properties of ECNFs can be optimized via template-free spinning or by refining spinning parameters and calcination conditions. The formation of bonded structures between ECNFs is a key enhancement strategy in three-dimensional reconstruction methods, wherein a larger interaction area enhances bond strength, and macroscale structural design optimizes mechanics through deformation characteristics of framework structures, which impose minimal constraints on building blocks while ensuring a degree of universality. Optimizing distinct mechanical properties necessitates tailored structural designs. Table 3 summarizes the mechanical properties of ECNFAs under various optimization strategies. In conclusion, the mechanical properties of ECNFAs can be enhanced through targeted optimization schemes or through integrating various optimization strategies, ultimately broadening the application scope of ECNFAs.

**Table 3:** Performance of ECNFAs with different Optimization Strategies

ECNFAs Type	Optimization strategies	Preparation method	Structure	Tensile (kPa)	Compressive (kPa)	Shear (kPa)	Bending (kPa)	Bucking (kPa)	Refs
Mullite/ α-Al_2_O_3_	Nanoscale dual-phase structure	Freeze-drying	Cellular	~ 35 at ε = 18%	~ 13 at ε = 80%	/	/	~ 16 at ε = 80%	[86]
Al_2_O_3_	Self-template	3D reactive electrospinning and freeze-drying	Lamellar	~ 120 at ε = 1.65%	~ 48 at ε = 80%	~ 650 at ε = 25%	/	~ 2.25 at ε = 50%	[95]
ZrO_2_	Self-template	Freeze-drying	Cellular	/	~ 2.4 at ε = 60%	/	/	/	[274]
SiO_2_	First all-ceramic aerogel	Freeze-drying	Cellular	~ 4.17 at ε = 3.5%	10.5 at ε = 80%	~ 6.79 at ε = 60%	/	/	[65]
SiO_2_	Structure design	Freeze-drying	Cellular interwoven	5.6 at ε = 10.7%	8.2 at ε = 85%	/		Bucking strain 5 ~ 85%	[64]
ZrO_2_-Al_2_O_3_	Structure design	Layer-by-layer stacking	Lamellar	/	1100 at ε = 90%	/	/	/	[68]
ZrO_2_‐TiO_2_	Structure design	Template receiving	Lamellar	/	15.87 at ε = 80%	/	/	/	[81]
holocrystalline zircon	Hypocrystalline and structure design	Blowing	Zigzag architecture	42.4 at ε = 18.5%	86.8 at ε = 95%	ε = 90%	1.08 at ε = 3.5%	/	[77]
Mullite	Structure design	3D reactive electrospinning	Interwoven interlocked	~ 12.7 at ε = 100%	~ 27.5 at ε = 80%	/	/	~ 2.25 at ε = 95%	[79]
TiO_2_	N-doped structure design	3D reactive electrospinning	Lamellar	17.3 at ε = 3.5%	43.91 at ε = 70%	/	/	/	[78]
ZrO_2_-SiO_2_	Structure design	3D reactive electrospinning	Interwoven interlocked	~ 45 at ε = 150%	35.3 at ε = 90%	torsion angle 360°	~ 8 at ε = 90%	/	[103]
Mullite/ Al_2_O_3_ microfibers	Microfiber and structure design	Freeze-drying	Bouligand chiral architecture	~ 170,380 at ε = 2.5%	156.47 at ε = 80%	/	/	/	[105]
SiO_2_	Structure design	Freeze-drying	Kirigami lamellated	33.6 at ε = 85%	~ 400 at ε = 80%		1cN at ε = 110%	~ 1.5 at ε = 60%	[104]
SiC	Bonding point construction	Freeze-drying	Cellular	/	~ 1.5 at ε = 55%	/	/	/	[53]
SiO_2_	Bonding point construction	Freeze-drying	Cellular	/	19.2 at ε = 80%	/	/	/	[275]
Al_2_O_3_	Bonding point construction	Freeze-drying	Cellular	/	11.3 at ε = 60%	/		/	[99]

## Application of ECNFAs

Ceramic aerogels assembled with flexible ECNFs have exhibited exceptional mechanical properties, including compressive resilience, stretch resilience, and high strength. These mechanical properties, combined with the structural characteristics of aerogels, such as high porosity, high specific surface area, and rich pore structure, show a wide range of application prospects. Thus, the section will discuss the structure–property relationships of ECNFAs and explore the application-specific mechanisms underlying these relationships and provide transformative solutions for advanced engineering applications.

### Thermal Management

ECNFAs integrate the intrinsic advantages of ceramic materials, including high-temperature resistance, low thermal expansion, high solar reflectivity, and strong mid-infrared emissivity, with the ultrahigh porosity (> 90%) and low density of aerogels, serving as versatile materials for advanced thermal management. By harnessing tailored combinations of ECNFs and functional architectures, ECNFAs can impede inward heat transfer or facilitate outward heat dissipation through various pathways, opening up opportunities for effective thermal management. Especially as insulating layers in equipment thermal protection systems or as cooling layers in industrial insulation systems, the ECNFAs can achieve effective thermal management by specifically blocking or emitting heat from various sources.

#### Heat Insulation

**Fig. 8:**
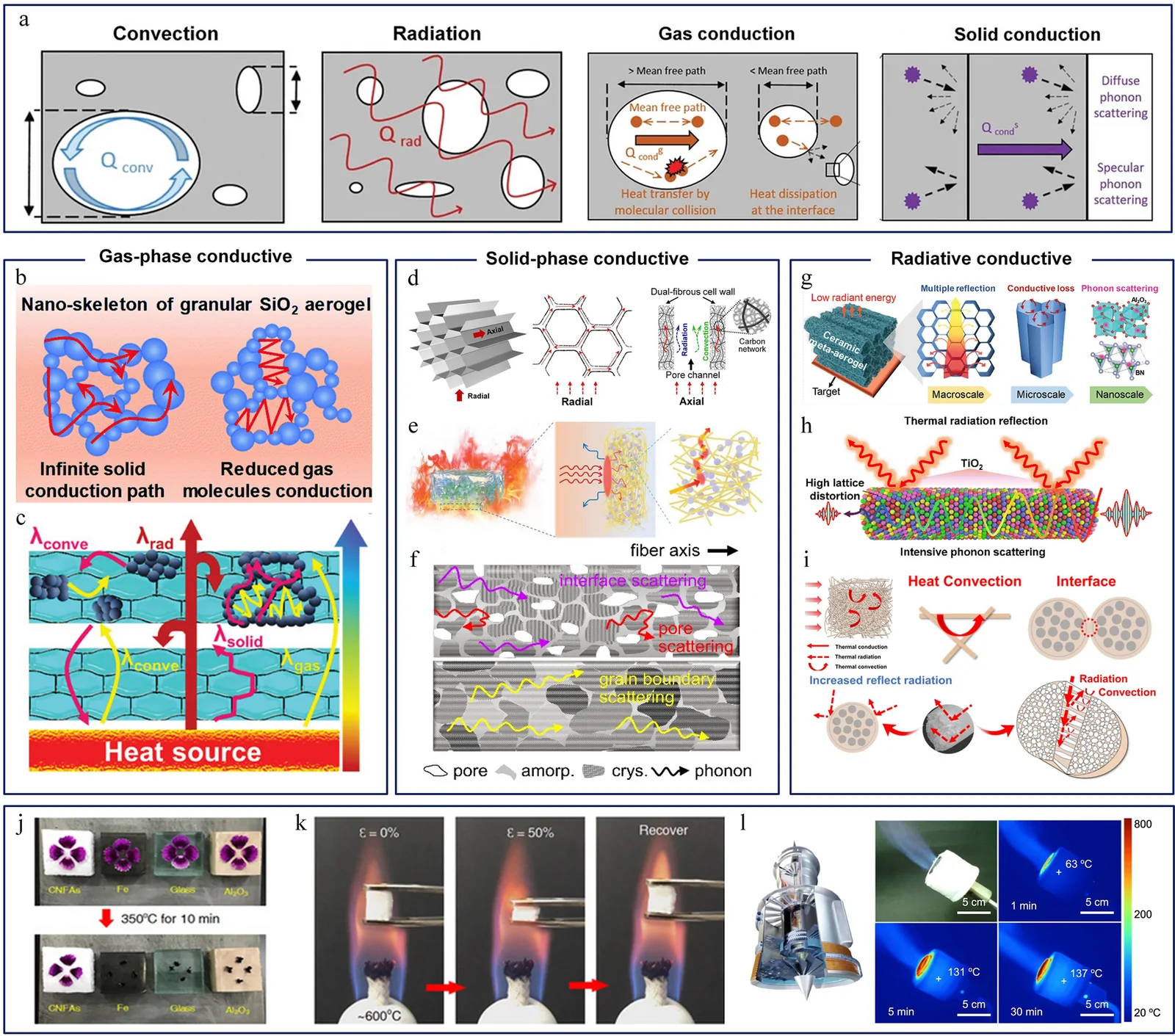
**a** Schematic representation of various modes of heat transfer. Adapted with permission from Ref. [290]. Copyright 2023, Wiley–VCH. **b** Schematic diagram illustrates heat conduction in a granular aerogel mesoporous structure. Adapted with permission from Ref. [112]. Copyright 2020, Royal Society of Chemistry. **c** Schematic depiction of heat transfer in a bilayer aerogel configuration. Adapted with permission from Ref. [95]. Copyright 2024, Wiley–VCH. **d** Schematic representation of heat transfer within a honeycomb-like cavity structure. Adapted with permission from Ref. [119]. Copyright 2022, Springer Nature. **e** Schematic diagram depicting the heat transfer pathway at a multiphase interface. Adapted with permission from Ref. [120]. Copyright 2023, Wiley–VCH. **f** Schematic illustration of phonon scattering at a heterogeneous interface. Adapted with permission from Ref. [117] Copyright 2023, Springer Nature. **g** Transmission mechanism of radiant heat in honeycomb structures. Adapted with permission from Ref. [99]. Copyright 2024, Wiley–VCH. **h** Inhibition mechanism of TiO_2_ sunscreen against thermal radiation. Adapted with permission from Ref. [123]. Copyright 2023, Royal Society of Chemistry. **i** Schematic illustration of radiative thermal conductivity within coaxial porous SiBCN/SiCN CNFAs structures. Adapted with permission from Ref. [124]. Copyright 2024, Elsevier. ECNFAs’s thermal insulation performance demonstration: **j** Optical images demonstrating the protection of flowers from burns using ECNFAs, **k** display of deformability of ECNFAs in the flame. Adapted with permission from Ref. [65]. Copyright 2018, American Association for the Advancement of Science. **l** Diagram illustrating the application of ECNFAs in thermal insulation. Adapted with permission from Ref. [79]. Copyright 2022, Springer Nature

Heat transfer within aerogels is quantitatively described by the superposition of conductive, radiative, and convective contributions (Fig. 8a). The total thermal conductivity (*λ*_*total*_) can be calculated as the following [1]:

4 $${\lambda }_{\mathrm{tot}}={\lambda }_{\mathrm{s}}+{\lambda }_{\mathrm{g}}+{\lambda }_{\mathrm{rad}}+{\lambda }_{\mathrm{con}}$$

where *λ*_*s*_, *λ*_*g*_, *λ*_*rad*_, *λ*_*con*_ represent the thermal conductivities of solid conduction, gas conduction, radiation, and convection, respectively. Among them, the *λ*_*con*_ usually is negligible for the pore sizes of aerogels less than 1 mm [106]. Generally, the pore architecture of ECNFAs is composed of three hierarchically organized components: micro-sized pores (10–30 μm) left by ice-template, nanofiber interwoven pore (1–2 μm) on the cell wall, and potential nano-sized pores in nanofibers. Thus, the structural configuration of ECNFAs restricts the primary thermal transfer mechanisms to solid-phase conduction, gas-phase conduction, and radiative heat transfer, while significantly diminishing the contribution of convective processes [107–109].

For the ECNFAs with high porosity, thermal energy primarily propagates via collisions between gas molecules and pore walls when exposed to a heat source. The magnitude of *λ*_*g*_ is governed predominantly by the pore structure [62], which can be quantitatively determined through the Knudsen effect model. The formula is displayed as follows [106]:

5 $${\lambda }_{g}=\frac{{\lambda }_{g}^{0}}{1+2\beta Kn}$$

6 $$Kn=\frac{{\Lambda }_{g}}{D}$$

where *λ*_*g*_^0^ denotes the intrinsic gas thermal conductivity, which is influenced by the bulk density of the aerogels. The dimensionless Knudsen number, *K*_*n*_, quantifies the ratio of the gas molecular mean free path (*Λ*_*g*_) to the characteristic pore diameter (*D*). The energy accommodation coefficient (β), reflecting the momentum exchange efficiency between gas molecules and pore walls, is highly dependent on surface chemistry and pore topology. According to Eqs. (5) and (6), *λ*_*g*_ in ECNFAs is critically determined by the ratio of *Λ*_*g*_ to *D*. For the ECNFAs with the porosity exceeding 90% directly constructed by the nanofibers, the micro-sized pores far exceed *Λ*_*g*_ (~ 70 nm for air at ambient conditions), which causes gas molecules to undergo frequent intermolecular collisions rather than interactions with pore walls, resulting in *λ*_*g*_ values of 0.024–0.037 W m^−1^ K^−1^ that marginally exceed that of stationary air (0.023 W m^−1^ K^−1^) [6]. To decrease *λ*_*g*_, the pore engineering strategies can transition the heat transfer regime from gas–gas collisions (continuum transport) to gas–wall collisions (Knudsen diffusion) [110]. Introducing mesopores (2–50 nm) in the aerogels reduces *D* to scales comparable with *Λ*_*g*_, elevating the Knudsen number and thereby amplifying the probability of gas–wall collisions, which imposes directional resistance to molecular motion, effectively scattering thermal energy and reducing *λ*_*g*_ [111]. For instance, incorporating a small amount of SiO_2_ aerogel particles with mesoporous networks (Fig. 8b) in the nanofiber framework can suppress *λ*_*g*_ to 0.02196 W m^−1^ K^−1^ [112, 113]. However, only a portion of the macropore structure is improved due to the limited load of SiO_2_ aerogel particles and the remaining unmodified macropores still provide significant pathways for gas conduction. To further decrease the *λ*_*g*_, a semi-closed pore structure (Fig. 8c) by introducing a large number of granular aerogels into the ECNFAs is designed, leading to a 16% reduction in *λ*_*tot*_ [68, 69, 95]. Although the semi-closed pore structure mitigates gas thermal conduction in open macropores, the approaches of partially introducing mesopores or fabricating semi-closed structures can merely attenuate gas conduction instead of eradicating it. Future progress requires fully closed mesopore structures to completely decouple the contributions of *λ*_*g*_, thus driving ECNFAs closer to the theoretical minimum thermal conductivity limit. In addition to regulating the macroporous structure of ECNFAs derived from ice crystal templates, the direct construction of meso-micropores with diameters smaller than the average molecular free path in the building blocks (ECNFs) can effectively restrict gas molecule movement and trap gas molecules within these pores, facilitating excellent thermal insulation performance. For example, creating a substantial number of mesopores with an approximate diameter of 18.9 nm in SiC fibers significantly reduces gas thermal conduction by trapping gas molecules [114].

*λ*_*s*_ plays an indispensable role in the thermal conductivity of the ECNFAs, which is mainly controlled by three factors: (i) the tortuosity of the nanofiber network, which elongates heat transfer pathways [115]; (ii) interfacial contact thermal resistance at nanofiber junctions [116]; (iii) intrinsic thermal conductivity of constituent nanofibers, dictated by phonon or atomic vibrational dynamics [117, 118]. When the ECNFAs are subjected to thermal gradients, heat propagates along the interconnected nanofiber skeleton, making the architectural configuration pivotal for modulating *λ*_*s*_. Dou et al. [119] engineered SiO_2_/C fiber aerogels with randomized honeycomb cells (Fig. 8d) via ice crystal-hydrogen bond co-assembly, utilizing structural tortuosity to extend heat transfer paths and achieve ultralow *λ*_*tot*_ (0.024 W m^−1^ K^−1^) across varying densities, while enabling anisotropic radial heat dissipation due to the anisotropy of the honeycomb structure. Concurrently, the interfacial thermal resistance at nanofiber junctions creates a bottleneck for continuous heat flow which can be reduced or modified by introducing heterogeneous interfaces. Hu et al. [120] embedded SiO_2_ nanoparticles into Al_2_O_3_ fiber aerogels (Fig. 8e) to create SiO_2_/Al_2_O_3_ interfacial barriers that disrupt phonon transmission, reducing *λ*_*tot*_ to 0.029 W m^−1^ K^−1^. When heat continues to be conducted along a single fiber, the intrinsic thermal conductivity of individual nanofibers is governed by intrinsic phonon transport in crystalline ceramics or atomic vibrations in amorphous systems. Taking crystalline ceramic nanofibers as an example, the governing equation for* λ*_*s*_ is expressed as follows [1]:

7 $${\lambda }_{\mathrm{s}}=\frac{1}{3}\rho {c}_{ph}{l}_{ph}{v}_{ph}$$

where *ρ*, *C*_*ph*_, *l*_*ph*_, and *v*_*ph*_ are the skeletal density, the volumetric heat capacity, the phonon mean free path, and the phonon group velocity (~ speed of sound), respectively. Minimizing *l*_*ph*_ via microstructural engineering, such as grain boundary scattering (Fig. 8f), defect introduction, or elemental doping can effectively reduce *λ*_*s*_. Guo et al. [77] synthesized hypocrystalline nanofibrous aerogels featuring multi-scale interface architectures through the strategic incorporation of polycrystalline ZrO_2_ nanoparticles (diameter < 10 nm) into an amorphous zirconia-based matrix. These interfaces intensified phonon scattering at crystalline-amorphous boundaries, slashing *l*_*ph*_ and achieving a low *λ*_*tot*_ (0.104 W m^−1^ K^−1^) at 1000 °C. In brief, the multi-scale strategies, optimizing nanofiber network tortuosity, enhancing interfacial thermal resistance, and engineering nanocrystalline-amorphous heterointerfaces, enable precise control over *λ*_*s*_.

*λ*_*rad*_ originates from the propagation of electromagnetic spectrum emissions at material boundaries where thermodynamic states exceed 0 K, with the radiative flux density being intrinsically governed by the object's inherent characteristics, such as its structure and emissivity. The typical formula about *λ*_*rad*_ is represented as follows [1]:

8 $$\begin{array}{c}{\lambda }_{\mathrm{rad}}=\frac{16}{3}{n}^{2}\sigma {T}^{3}/(e\cdot \rho )\end{array}$$

where *n*, *σ*, *T*, *e*, and *ρ* represent the refractive index, the Stefan-Boltzmann constant, temperature, emissivity, and density, respectively. When thermal radiation is transmitted through ECNFAs, the multi-scale framework structure of ECNFAs first hinders radiation transmission through reflection and absorption. Therefore, a well-designed structure can provide primary thermal radiation protection for ECNFAs. A representative example is the interlocked honeycomb structure formed by the interweaving of BN/C/Al_2_O_3_ fibers, wherein interwoven honeycomb macroscale cells provide multiple reflections and absorption losses for thermal radiation and interlocked nanofiber interfaces offer numerous microscale interfaces for thermal radiation reflection (Fig. 8g). The multi-scale structure design results in an excellent suppression of radiation heat transfer, allowing the ECNFAs to limit the backside temperature to 135 °C even in a 1300 °C thermal field, demonstrating significant infrared stealth potential [99]. As thermal radiation propagates further through the framework, the nanofiber structure becomes an effective means to suppress it. Generally, the most effective and simple method is to load high-reflectivity additives (such as TiO_2_ and carbon) on the surface of nanofibers [121, 122]. For instance, embedding TiO_2_ opacifiers into medium-entropy ECNFs (Fig. 8h) can double the infrared reflectivity of the ECNFs, thereby significantly reducing the thermal conductivity of ECNFAs to 0.089 W m^−1^ K^−1^ at 1000 °C [123]. Beyond adding the high reflectivity additives, geometric engineering of ECNFs can introduce hierarchical photon-trapping architectures to decrease *λ*_*rad*_. For example, coaxial porous structures (Fig. 8i), comprising alternating dense and porous layers, expand the inhibitory path via multi-scale reflections. Wang et al. [124] fabricated SiBCN/SiCN coaxial nanofibers with nested porosity to achieve a thermal conductivity of ECNFAs of 0.125 W m^−1^ K^−1^ at 1200 °C, outperforming homogeneous analogs by 40%. The mechanism arises from (i) interface scattering at coaxial boundaries, (ii) photon confinement within nanoscale pores, and (iii) resonance absorption by mid-infrared-active chemical bonds (e.g., Si–C, B–N). Such designs amplify the "multiple reflection effect" of ECNFs on thermal radiation, trapping the radiant heat energy inside the ECNFs and preventing its transmission [125].

Due to the unique porous structure of ECNFAs, heat is transferred within them through interdependent mechanisms. While traditional strategies, such as pore engineering for gas conduction suppression, phase/interface optimization for solid conduction reduction, and opacifier integration for radiative attenuation, individually target specific heat transfer pathways, their isolated application fails to achieve comprehensive thermal management. To overcome the limitation, synergistically decoupling gas, solid, and radiative heat transfer through different methods has emerged as a promising paradigm [126, 127]. A pioneering demonstration by Chang et al. [128] integrated carbon and nano-sized mullite-based ceramic compounds into individual 1D nanofibers and assembled these nanofibers into 3D elastic aerogels with a layered honeycomb and locally closed-cell structure. In the ECNFAs, gas conduction is minimized by sol-derived closed-pore films that restrict molecular gas movement; solid conduction is attenuated via carbon/ceramic interfacial phonon scattering; radiative transfer is suppressed through multiphase solid–gas interfaces that reflect, refract, and absorb infrared radiation, which is augmented by the carbon component’s inherent infrared shielding capability. The multi-mechanism coordination effectively intercepts all dominant heat transfer pathways, endowing the ECNFAs with ultralow thermal conductivity (e.g., 0.051 W m^−1^ K^−1^ at 300 °C) and exceptional thermomechanical stability (e.g., 1600 °C in oxidative environments) (Fig. 8j–k). Such hierarchical engineering establishes ECNFAs as next-generation solutions for extreme-environment insulation in aerospace and energy systems (Fig. 8l), where simultaneous thermal and mechanical stability are paramount.

As summarized in Table 4, these various strategies, spanning pore structure control, interfacial phonon engineering, and radiative suppression, provide a roadmap for advancing ECNFAs as next-generation thermal insulators, balancing extreme-temperature performance with structural resilience.

**Table 4:** Comparison of thermal insulation properties of different types of ECNFAs

ECNFAs type	Optimization Strategies	Density (mg cm^−3^)	Thermal conductivity (W m^–1^ k^–1^, room temperature)	Work temperature (℃)	Refs
SiO_2_	Interweaved cellular structure	0.5	0.0223	1100	[64]
	kirigami lamellated structure	1	0.033	1100	[104]
	tortuous channels structure	4	0.024	1100	[275]
	SNAs and SNF	7.69	0.02196	1000	[112]
	First all-ceramic fiber aerogel	10	0.025	1100	[65]
	laminated structure and MMT	50	0.0397	1100	[276]
SiO_2_/SnO_2_	SnO_2_ doped	0.093	0.025	800	[277]
SiO_2_/Al_2_O_3_	Al-doped	6.21	0.022	1000	[278]
ZrO_2_-SiO_2_	hypocrystalline	5 ~ 20	0.0254 0.1046 at 1000 °C	1000	[103]
	SGA, ZrO_2_–SiO_2_ NF and Lamellated structure	23	0.024	1100	[69]
ZrO_2_	Fiber intrinsic insulation	10	0.028	1300	[274]
hypocrystalline zircon	hypocrystalline	20	0.026 0.104 at 1000 °C	1300	[77]
ZrO_2_‐TiO_2_	TiO_2_-doped	9.5	0.027	1200	[81]
Al_2_O_3_	SAM and Al_2_O_3_ NF	0.01	0.029	1300	[120]
	SNAs and Al_2_O_3_	22	0.027	1700	[95]
Aluminum borate	Fiber intrinsic insulation	276	0.11	1200	[115]
Al_2_O_3_/ZrO_2_	hypocrystalline	3.4	0.0216	1500	[117]
Mullite	Fiber intrinsic insulation	6	0.0228	1300	[79]
	mullite nanosheets and NFs	18	0.028	1300	[279]
	Multiphase symbiotic engineered	30	0.051 at 300 °C	1600	[128]
Mullite/α-Al_2_O_3_	Multiphase ceramic	10	0.032	1600	[86]
Mullite/Al_2_O_3_ microfibers	Microfiber and structure design	223	0.037, 0.076 at 300 °C	1200	[105]
TiO_2_	Fiber intrinsic insulation	12	0.0285	800	[78]
(Ti_0.42_Zr_0.42_Y_0.08_Si_0.08_)O_2.08_	TiO_2_-doped	10	0.089 at 1100	1400	[123]
SiC	Fiber intrinsic insulation	4.84	0.019	1300	[53]
SiBCN	TiO_2_-doped	/	~ 0.024 at 1300 °C	1300	[121]
SiBCN/SiCN	Coaxial porous SiBCN/SiCN ceramic fiber	0.12	0.031 0.125 at 1000 °C	1300	[124]
SiZrOC	Zr-C-doped	/	0.024 0.092 at 800 °C	1300	[116]
ZrC	Fiber intrinsic insulation	0.0133	0.185	1400	[126]

#### Radiative Cooling

In daily environments, the excess heat generated by solar radiation increases energy consumption and exacerbates greenhouse gas emissions. Consequently, effective management of the surplus solar heat is crucial for energy conservation and environmental protection. Passive radiative cooling has emerged as a sustainable strategy to mitigate energy consumption and greenhouse emissions by dissipating excess solar heat through the atmospheric transparency window (ATW: 8–13 μm) [129]. Conventional materials to achieve selective ATW emissions (such as ZnS or ZnSe [130, 131] or HfO/SiO_2_ composites [132]) often rely on costly, mechanically fragile micro/nanopatterned structures, limiting their practical scalability [130, 132–134]. ECNFAs circumvent these challenges by synergizing intrinsic ceramic optical properties, aerogel-derived ultrahigh porosity, and nanofiber-enabled flexibility. For instance, compressible silica-alumina nanofiber aerogels leverage nanofibers and micropores for broadband solar reflectance (95%) and ceramic phonon-polaritons for enhanced mid-infrared emissivity (93% within ATW), yielding an average radiative cooling power of 133.1 W m^−2^ and sub-ambient temperature reductions exceeding 5 °C [69]. Advancing beyond static performance, dual-mode ECNFAs enable dynamic thermal regulation via redox-driven spectral modulation. For example, the white TiO_2_ nanofiber sponges (WTNS) exhibit high solar reflectance (0.89) and ATW emissivity (0.91), cooling surfaces by 11 °C, while their carbon-reduced counterparts (BTNS) switch to high solar absorption (0.92) and emissivity (0.94), heating surfaces by 13 °C. The spectrally adaptive behavior, combined with mechanical resilience (90% compressive strain recovery) and scalable electrospinning synthesis, positions ECNFAs as transformative materials for intelligent thermal management systems, enabling environment-responsive thermal regulation in applications ranging from smart textiles to energy-efficient buildings.

In summary, researchers have significantly enhanced the thermal insulation properties of ECNFAs via multi-scale engineering strategies: atomic-scale phase engineering optimizes phonon transport, micrometer-scale pore regulation suppresses gas conduction, mesoscopic-scale heterointerfaces and filler doping enhance phonon scattering and macroscopic-scale fiber assembly designs extend heat transfer pathways. Simultaneously, these innovations, combined with the tunable optical properties of ceramics, enable dynamic thermal management—achieving simultaneous radiative cooling and heating in a single material system. Despite progress, the operational stability of ECNFAs above 1300 °C remains limited by nanofiber degradation and structural collapse under prolonged thermal stress. Future research must prioritize the development of ECNFAs that can stably operate at elevated temperatures. Additionally, the unique infrared transparency and emissivity of ECNFAs position them as ideal candidates for radiative cooling. It is expected that ongoing research will expand their functional applications and drive the development of thermal management materials.

### Environmental Field

ECNFAs exhibit significant application potential in environmental engineering due to their multi-scale structures and programmable functionalization. By strategically selecting constituent materials (e.g., TiO_2_ for catalytic activity [135], SiC for electromagnetic absorption [136]) and engineering hierarchical architectures, including surface-functionalized nanofibers, tailored meso-/macroporous networks with specific grades, and designed framework structures, ECNFAs can achieve multifunctional capabilities such as high-efficiency filtration, adsorption–separation, catalytic degradation, and electromagnetic shielding. Meanwhile, the structural-mechanical coupling of the nanofiber skeleton ensures structural stability under dynamic operational stresses. These attributes, combined with their ultrahigh surface area and tunable active sites, position ECNFAs as a core material for addressing global complex environmental challenges.

#### Gas Purification

Air, an indispensable element for human survival, has faced escalating contamination due to modern industrialization, with airborne pollutants, including pathogenic microorganisms (bacteria, viruses), toxic gases (volatile organic compounds [VOCs], hazardous chemical agents, and carbon monoxide [CO]) posing severe health risks. Effective air purification is thus critical to safeguarding public health, driving demand for advanced materials that combine high filtration efficiency with low operational resistance. Conventional porous membranes (e.g., polytetrafluoroethylene (PTFE) [137], polyethylene (PE) [138], and polypropylene (PP) [139]) and fibrous membranes (e.g., Al_2_O_3_ [140] and SiC [141]) exhibit limitations such as high filtration resistance and low adsorption capacities, restricting their practical application. In contrast, ECNFAs offer a breakthrough solution owing to their three-dimensional interconnected pore networks, ultrahigh specific surface area, and customizable pore structure. Based on the existing research work, the application in gas purification of ECNFAs can be broadly categorized into microbial inactivation and detoxification of toxic gases.

**Fig. 9:**
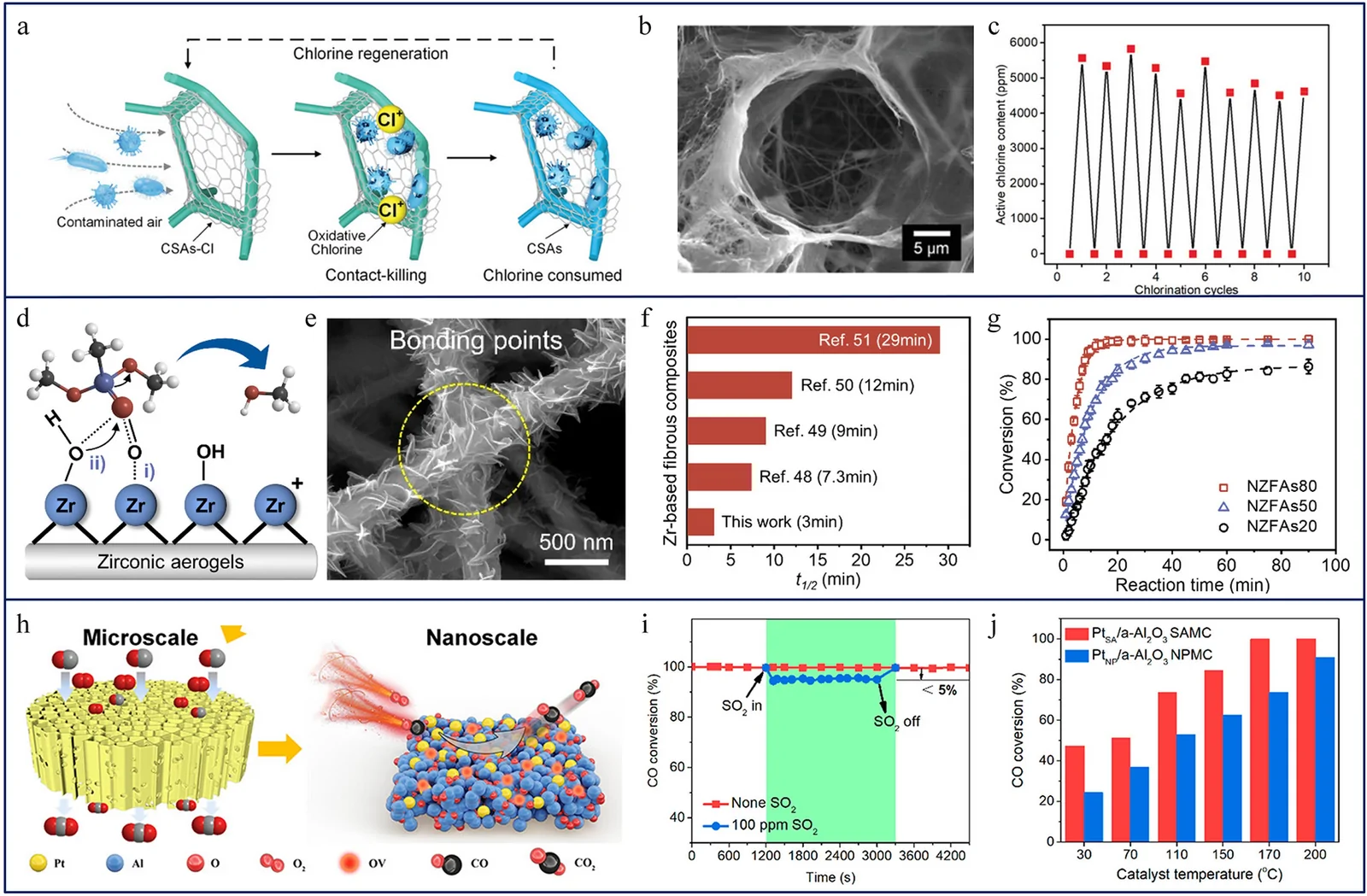
**a** Sterilization mechanism, **b** SEM image, and **c** the active chlorine content after cyclic chlorination of the dual-network aerogel. Adapted with permission from Ref. [142]. Copyright 2021, Wiley–VCH. **d** Degradation mechanism, **e** SEM image, **f** the half-life contrast, and **g** the curve of DMMP conversion versus reaction time of Zr (OH)_4_ nanosheet-based ECNFAs. Adapted with permission from Ref. [147]. Copyright 2021, American Chemical Society. **h** CO oxidation mechanism, **i** CO oxidation efficiency, and **j** the high durability of Pt_SA_/a-Al_2_O_3_ nanofibrous aerogels. Adapted with permission from Ref. [149]. Copyright 2023, American Chemical Society

For protection against airborne microorganisms, highly efficient filtration materials are essential in daily life. Among these, nanofiber membrane materials are favored for their dense structure and high filtration efficiency. However, the dense structure also results in a high-pressure drop and significant energy consumption. Unlike traditional ECNMs, ECNFAs leverage the bridging of nanofibers to form three-dimensional interconnected pores, enabling high gas permeability. However, they have limited interception efficiency for submicron contaminants (e.g., bacteria: 0.5–5 μm; viruses: ~ 0.3 μm) and risks of microbial proliferation. The problems can be solved by refining the mesh structure, such as constructing secondary pores on the macropore (10–30 μm) structure of ECNFAs, while the risk of microbial proliferation can be addressed by endowing ECNFAs with antibacterial functions. For example, integrating bacterial cellulose (BC) nanofibers into a SiO_2_ framework can create a hierarchical dual-network structure with 200-nm secondary pores nested within macropores (Fig. 9a, b). The nano-to-macro-porosity enhanced diffusion-capture interactions, achieving > 99.97% filtration efficiency for 0.3 μm particles at an ultralow-pressure drop (189 Pa). Meanwhile, *N*-halamines were covalently grafted onto SiO_2_ nanofiber framework via sol–gel processing, endowing ECNFAs with rapid, regenerable antimicrobial activity, making ECNFAs achieve a 6-log reduction of E. coli within 3 min and of bacteriophages within 5 min, and stable performance over 10 chlorination cycles (Fig. 9c) [142].

Currently, the main approach to toxic gas detoxification is to utilize porous materials with high specific surface areas, such as activated carbon [143, 144] and zeolites [145, 146], which absorb the gases followed by post-treatment processes like thermal or chemical regeneration to break them down. However, the approach involves lengthy processing steps, high energy consumption, and a risk of secondary pollution. In contrast, directly loading catalysts with catalytic degradation capabilities, such as metal hydroxides (e.g., Zr(OH)_4_ [147]), metal–organic frameworks (MOFs) [148], and single-atom catalysts (e.g., Pt) [149], is more efficient and safer. Additionally, unlike the low porosity and short penetration channels of traditional densely packed two-dimensional fiber composites, the three-dimensional interconnected nanofiber framework of ECNFAs not only offers a large number of loading sites but also enables the rapid diffusion of toxic gases within the framework, which effectively promotes the detoxification of these gases. For example, vertically aligned Zr(OH)_4_ nanosheets grown on ECNFAs via 3D spatial confinement (Fig. 9d, e) accelerate the degradation of organophosphates like DMMP, achieving a half-life (t_1/2_) of 3 min and 99% DMMP conversion within 20 min (Fig. 9f, g) [147]. However, mechanical detachment of these catalytic units under operational stress compromises long-term service performance. Yan et al. [148] anchored MOF-808 to SiO_2_ nanofiber skeleton via hydrogen-bond networks formed by the ceramic sol interface to ensure structural integrity (5% plastic deformation after 1000 compression cycles) and stable viscoelasticity (constant storage/loss moduli across 0.01–10 Hz). The anchored MOF-808 conferred a dynamic DMMP processing capacity of 400 L g^−1^, demonstrating potential for industrial deployment. Another challenge during the introduction of catalytic units is the agglomeration of atomic-scale catalysts, which can be addressed by the Kirkendall effect that allows two metals with different diffusion rates to form voids at the interface during interdiffusion, enabling the independent dispersion of single-atom catalysts. For example, during the calcination of Pt-anchored Al–O–Al gel precursors, rapid outward diffusion of Pt ions and slower inward diffusion of Al^3+^ ions creates interfacial vacancies, driving Pt migration to the nanofiber surface. The process stabilizes Pt as isolated single atoms on amorphous alumina (Pt_SA_/a-Al_2_O_3_) nanofibers and results in Pt_SA_/a-Al_2_O_3_ nanofibrous aerogels (Fig. 9h) with exceptional CO oxidation activity, achieving 100% conversion at 170 °C (Fig. 9i, j) [149]. However, the method of directly loading catalytic degradation functional catalysts still has some flaws, such as the limited-service life of the catalysts and low load. Therefore, the enhancement of the intrinsic catalytic performance of ECNFs is a crucial future direction for ECNFAs-based toxic gas purification materials. TiO_2_/GO nanofiber aerogels degraded DMMP at 0.115 min^−1^ under photothermal conditions, outperforming conventional TiO_2_ composites by 57% [150]. Despite progress, challenges persist in intercepting ultrafine particulates (< 200 nm), degrading volatile organic compounds (VOCs), and expanding intrinsic ceramic catalysis (e.g., ZnO, TiO_2_) without external modifiers. Future research must further refine the pore structure, the expansion of functions, and the development of intrinsic catalytic effects to advance ECNFAs toward next-generation air purification systems.

#### Water Treatment

The discharge of pollutants, including pathogenic microorganisms, organic compounds, and oils, into aquatic systems poses severe risks to environmental and human health. To address the challenges, advanced materials such as activated carbon [151, 152], ion-exchange resins [153, 154], ECNMs [155, 156], and ECNFAs [157–160] have been developed for water remediation. Among these, ECNFAs stand out due to their exceptional chemical stability, ultrahigh specific surface area, tunable hierarchical porosity, and facile surface functionalization enabling multifunctional applications in water disinfection [161], adsorption–separation and degradation of organics [162–166], and desalination [67, 100, 163, 167, 168].

**Fig. 10:**
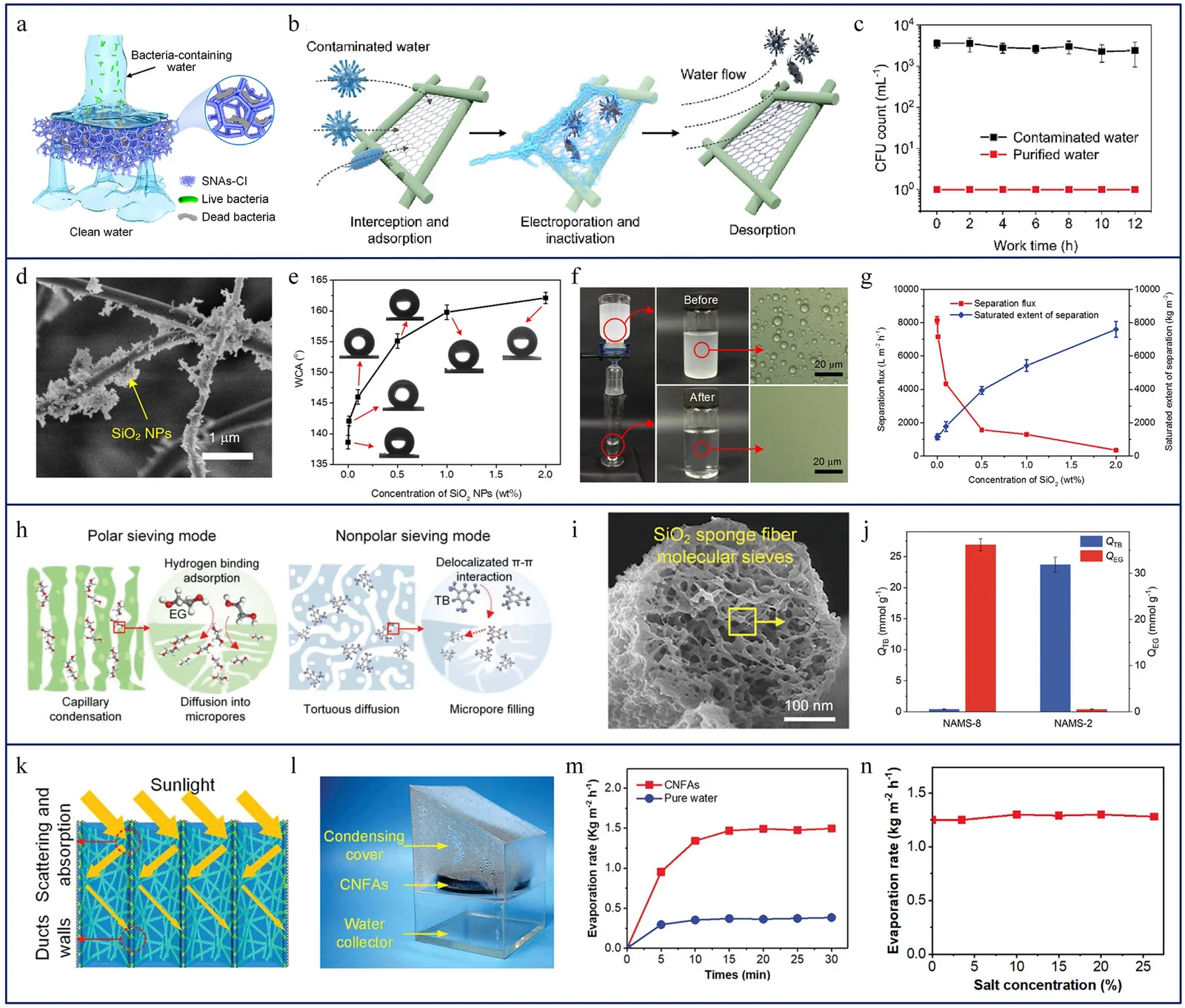
**a** Schematic diagram of ECNFAs filtration sterilization. Adapted with permission from Ref. [161]. Copyright 2020, American Chemical Society. **b** Sterilization mechanism and **c** water purification effect of SiO_2_/Ag nanowire aerogels. Adapted with permission from Ref. [169]. Copyright 2023, Elsevier. **d** SEM image, **e** WCA, **f** water-in-oil emulsions separation pictures, and **g** oil–water separation efficiency of ECNFAs loaded with SiO_2_ nanoparticles. Adapted with permission from Ref. [179]. Copyright 2015, American Chemical Society. **h** Nano-gating effect, **i** SEM image, and **j** separation effect of NAMS. Adapted with permission from Ref. [159]. Copyright 2022, Wiley–VCH. **k** Schematic diagram of solar energy absorption mechanism, **l** solar evaporator picture, and the evaporation rate varies with** m** time and of CNTs@SiO_2_ nanofibrous aerogels. Ref. [157]. Copyright 2020, Wiley–VCH. **n** Stability of ECNFAs with vertically aligned parallel pipe walls under different salt concentrations. Adapted with permission from Ref. [158]. Copyright 2021, American Chemical Society

*Water Disinfection* Water disinfection demands materials that synergize rapid microbial inactivation with structural stability under high hydraulic fluxes, posing distinct challenges compared to gas-phase purification. ECNFAs with high porosity, structural stability, and mechanical robustness are preferred for rapid-mode sterilization mechanisms. Bifunctional silane-based binders that incorporate *N*-halamine precursors and a “rigid-flexible synergy” cross-linking network enable simultaneous antimicrobial functionalization and robust structural engineering. The hybrid network, formed via covalent Si–O-Si bonds and hydrogen bonding, endows ECNFAs with mechanical robustness (30.7 kPa compressive strength at 80% strain) and high permeability (57,600 L m^−2^ h^−1^ at 20 kPa), while facilitating effective sterilization (Fig. 10a). Hydrophilic SiO_2_ nanoparticles were introduced to further enhance surface wettability (Fig. 9a), ensuring rapid water spreading to maximize pathogen contact with *N*-halamine sites, achieving a 6-log reduction of E. coli and S. aureus (99.9999% inactivation) [161]. However, chemical-dependent biocides carry the risk of depletion and secondary toxicity. To overcome these limitations, physical sterilization methods, such as electroporation, offer a promising solution. In ECNFAs, the technique can be implemented by introducing a secondary conductive network with densely distributed nano-tips into the ceramic nanofiber framework (Fig. 10b). For example, introducing Ag nanowires into the SiO_2_ nanofiber framework creates conductive nano-tip arrays (Fig. 10c) to generate localized electric fields (~ 10^7^ V m^−1^) at ultralow voltages (1–5 V), which induces bacterial membrane electroporation, achieving > 6 log removal of bacteria and > 3 log inactivation of virus, with sustained efficacy over 12 h and negligible energy consumption (0.83 Wh m^−3^) [169]. Unlike chemical methods, the approach eliminates secondary pollution risks and maintains performance under continuous flow, ideal for industrial wastewater treatment.

*Adsorption Separation and Degradation of Organics* The increasing contamination of water resources by organic pollutants such as oils, solvents, and dyes calls for advanced materials that can perform selective adsorption, separation, and catalytic degradation [170]. Conventional materials, like activated carbon [171], polymer resins [172], and separation membranes [173, 174], face limitations in porosity, diffusion kinetics, and stability, often leading to secondary pollution [175]. ECNFAs offer a robust solution due to their ultrahigh porosity, tortuous interconnected pores, and adaptable surface characteristics. Oil pollution is one of the most representative water pollutants, and surface-engineered ECNFAs achieve superior selectivity through tailored selective oil/water affinity compared to traditional membrane separation materials [162, 176–178]. Integrating SiO_2_ nanoparticles creates nanoscale roughness (Fig. 10d), inducing a Cassie–Baxter state for superhydrophobicity (water contact angle: 162°) (Fig. 10e), while lipophilic polymers (e.g., PBZ, PVDF) impart near-zero oil contact angles. The synergy enables gravity-driven separation of oil-in-water emulsions with 99.995% purity and an adsorption capacity of 7612 ± 480 kg m^−3^, sixfold higher than conventional membranes (Fig. 10f, g) [179]. Photothermal graphene oxide (GO)-functionalized ECNFAs further enhance crude oil recovery by reducing viscosity via solar-driven heating, slashing absorption time by 99.65% (300 s) (Fig. 9h) [180]. Similarly, surface engineering of ECNFAs through carboxylation [165] or phosphorylation [181] introduces charged functional groups (–COO^−^, –PO_4_^3−^), enabling selective adsorption of positively charged proteins via electrostatic interactions while repelling negatively charged species to achieve efficient separation of proteins. Polarity-driven surface engineering further broadens the application scope of ECNFAs, extending from oil–water and protein separation to organic solvent purification. Biomimetic nanofibrous aerogel molecular sieves (NAMS) combine polar silica and nonpolar PS/PVDF fibers to achieve sub-nanometer gating effects (Fig. 10h, i). Polar NAMS-8 adsorbs ethylene glycol (36.2 mmol g^−1^) via hydrogen bonding, while nonpolar NAMS-2 captures trimethylbenzene (23.7 mmol g^−1^) through π–π interactions (Fig. 10j) [159]. In addition to surface functional modification, when ECNFs with intrinsic catalytically active sites on the surfaces are constructed into ECNFAs, they demonstrate the capability for dye adsorption and degradation. CeO_2_/SiO_2_ core-sheath aerogels utilize Ce^3+^/Ce^4+^ redox pairs, wherein Ce^3+^ coordinates with RhB’s amino groups (218.5 mg g⁻^1^ adsorption), while Ce^4+^ oxidizes chromophores [164]. These multifunctional designs underscore ECNFAs’ versatility in addressing complex pollution challenges while aligning with sustainable development goals.

*Desalination* Beyond the aforementioned types of water treatment for pollution, the development of energy-efficient desalination technologies is critical to addressing global freshwater scarcity. Solar photothermal desalination emerges as a sustainable strategy by leveraging solar energy for seawater evaporation [182]. It relies on photothermal materials to convert solar radiation into heat [167, 183, 184], driving surface water evaporation. However, heat loss and salt accumulation during evaporation remain critical challenges. ECNFAs with hierarchical porosity, ultralow thermal conductivity, and tunable structural architectures are the optimal materials to address the above issues. For instance, honeycomb-like networks in ECNFAs constructed from SiO_2_ nanofibers and CNTs (CNTs@SiO_2_ nanofibrous aerogels) can enhance solar energy utilization efficiency via repeated internal reflection and scattering of the CNT cavity wall (Fig. 10k) while maintaining the least heat loss. The solar evaporator that utilizes CNTs@SiO_2_ nanofibrous aerogels is capable of achieving 98% broadband solar absorption within the wavelength range of 200 to 2500 nm and an evaporation rate of 1.50 kg m^−2^ h^−1^ within 15 min (Fig. 10l, m) [157]. In addition to improving the utilization rate of solar energy, the design of vertically aligned parallel pipe structures can effectively minimize the diffusion path, allowing the salt to quickly return to the seawater body, alleviating salt fouling issues, such as vertically aligned Janus MXene-based aerogels [168, 183]. Even with optimal structural design, a small amount of salt residue remains, which is not conducive to the long-term use of solar evaporators. To address the problem, inspired by the structure of reed leaves, hydrophobic modification of vertically aligned parallel pipe walls suppresses surface crystallization, sustaining stable operation in saturated brine (26.3 wt% NaCl) for > 6 h (Fig. 10n), outperforming conventional evaporators [158]. However, in seawater contaminated with oil or organic matter, the effectiveness of these designs has significantly declined. Therefore, designing multifunctional solar evaporators is crucial for evaporating contaminated seawater. Functional modification strategies such as structural design and the introduction of functional units also provide feasible paths for designing multifunctional solar evaporators. A representative example is the design of multifunctional aerogels (FMAs) with layered (l-FMA) and hierarchical honeycomb (c-FMA) structures, fabricated from MXene-wrapped ECNFs, wherein c-TNFs enable gravity-driven oil–water separation (Flux: 1,100 L m^−2^ h^−1^; TOC < 10 mg L^−1^), while l-TNFs attain 93.5% solar absorption and 1.482 kg m^−2^ h^−1^ evaporation (92.08% efficiency) via light-trapping MXene interfaces [67]. Similarly, the integration of efficient photothermal materials with functional materials designed for degradation and adsorption offers a viable solution for the purification and evaporation of dye-contaminated seawater. For example, Co-doped MoS_2_/γ-Al_2_O_3_ aerogels, where Co_x_Mo_1-x_S_2_ achieved high photothermal conversion efficiency, exhibited a seawater evaporation rate of 1.62 kg m^−2^ h^−1^. Meanwhile, the protonated surface promoted by the abundant hydroxyl groups in γ-Al_2_O_3_ facilitates dye purification through electrostatic adsorption and nucleophilic degradation [163]. Additionally, Multifunctional MOFs, such as ZIF-8 [185], UiO-66 [186], NU-1000 [187], and MOF808 [188], further expand the functionality of comprehensive water purification [189].

In summary, ECNFAs have demonstrated significant potential in water treatment, but more challenges persist. Current ECNFAs predominantly rely on external functionalization units, such as MOF integration or single-atom catalysts integration to enhance performance. Therefore, future efforts should focus on utilizing the intrinsic physicochemical properties of ECNFs to develop high-performance functions without dependency on secondary functional units. Meanwhile, as water treatment scenarios grow increasingly complex, spanning multi-pollutant systems and dynamic environmental conditions, the development of multifunctional ECNFAs is imperative. For example, enabled by hierarchical designs and tailored surface chemistry, synergizing adsorption, catalysis, and antimicrobial activity within a single ECNFA. Furthermore, scalability, cost-efficiency, and long-term stability under operational stress also need to be addressed.

#### Noise Control

**Fig. 11:**
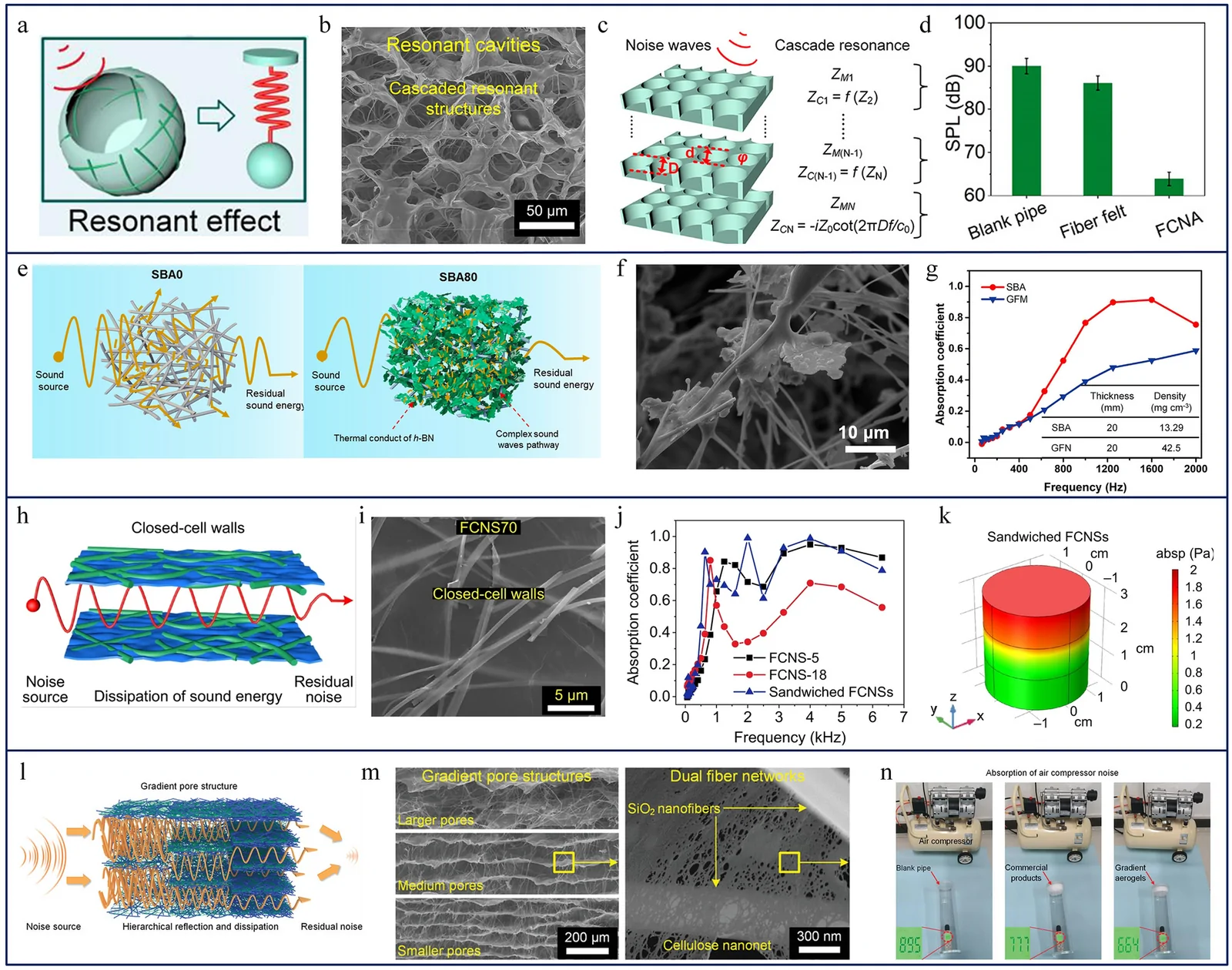
**a** Resonant effect, **b** SEM image, **c** cascade mechanism for acoustic absorption, and **d** noise absorption effectivity of ECNFAs with resonant cavity structures. Adapted with permission from Ref. [73]. Copyright 2022, American Chemical Society. **e** acoustic absorption mechanism, **f** SEM image, and **g** noise absorption effectivity of SBAs. Adapted with permission from Ref. [196]. Copyright 2022, American Chemical Society. **h** Sound absorption mechanism, **i** SEM image, **j** sound absorption effectivity, and **k** sound pressure distribution simulation of sandwich-structured ECNFAs with the closed cell wall. Adapted with permission from Ref. [101]. Copyright 2021, Springer Nature. **l** Acoustic absorption mechanism, **m** SEM image, and** n** noise-absorbing test of gradient structural aerogel. Adapted with permission from Ref. [197]. Copyright 2023, Wiley–VCH

Noise pollution poses a significant threat to human health and environmental quality, necessitating advanced solutions for sound attenuation. The strategies for reducing noise, such as minimizing noise generation at the source, using sound-absorbing materials during propagation, and wearing personal protective equipment at the point of reception, are widely adopted. Among these strategies, sound-absorbing materials are especially notable due to their simplicity and effectiveness [190]. Based on their absorption mechanisms, sound-absorbing materials can be categorized into two main types: resonant absorbers and porous absorbers. Resonant absorbers, such as ECNFAs, dissipate energy via frequency-dependent resonance, excelling in mid-to-low-frequency ranges (200–1000 Hz) [73]. For instance, cascaded resonant cavities engineered within ECNFAs through bubble-assisted freeze-casting (Fig. 11a, b) exploit synergistic interactions between multilayer series acoustic impedance (thickness direction) and parallel acoustic impedance from unit cavities (in-plane direction), generating a cascaded resonance effect (Fig. 11c). The design achieves exceptional low-frequency absorption while maintaining stability under extreme conditions, reducing air compressor noise from 90.0 to 63.9 dB (Fig. 11d) [73].

Unlike resonant absorbers, porous sound-absorbing materials attenuate noise through viscous friction and thermal dissipation, converting acoustic energy into heat across a broad frequency spectrum [190, 191]. The earliest porous aerogel materials used for sound absorption were polymer-based aerogels which achieved moderate absorption (NRC ≤ 0.51) via tortuous macroporous channels [192–194], but they had thermal instability (> 150 °C degradation) and limited efficacy in low-frequency regimes (0–2000 Hz), where penetrating sound waves bypass macroporous structures [101]. To address these challenges, ECNFAs with exceptional thermal resilience (> 500 °C) and tunable pore architectures are designed and developed [195]. Recent studies reveal that pore size critically governs the acoustic performance of porous absorbers, wherein large pores enhance high-frequency noise dissipation, while small pores optimize low-frequency noise absorption [192]. Therefore, to enhance the sound absorption effect of ECNFAs in the low-frequency range, structural engineering is carried out using h-BN nanosheets to segment macropores within SiO_2_ nanofiber aerogels (SBAs) into hierarchical frameworks (Fig. 11e, f), which can prolong sound wave paths and enhance low-frequency absorption (0–2000 Hz, NRC ~ 0.59) with ultralow density (13.29 mg cm^−3^, Fig. 11g) [196]. However, the method of macro-pores segmentation of ECNFAs compromises high-frequency absorption. A straightforward and versatile approach is to directly combine ECNFAs with both high and low-frequency sound absorption capabilities. The effective cases are to construct the ECNFAs with a sandwich-layered structure, which is achieved by combining layered ECNFAs with "low-density/medium-density/low-density" configurations. The different volume densities of ECNFAs create a "high-frequency/low-frequency/high-frequency" broadband dissipation composite layer along the thickness direction. Within each layer, the open cell walls, semi-open cell walls, and closed pore walls, mediated by GO nanosheets (Fig. 11h, i), efficiently dissipate the incident sound waves, resulting in the sandwich-layered ECNFAs with excellent ultra-wideband noise absorption performance (63–6300 Hz) and an NRC of approximately 0.56 (Fig. 11g–k) [101]. However, the high density of the middle layer reflects high-frequency waves, limiting their penetration into subsequent layers. To address the issue, a gradient pore architecture (“large pores/medium pores/small pores” along the thickness direction) was engineered, enabling stepwise dissipation of incident waves (Fig. 11l, m). By progressively channeling sound energy through decreasing pore size, the gradient structure minimizes reflection losses and maximizes broadband absorption (NRC ~ 0.58), realizing the decrease of air compressor noise from 89.5 to 66.4 dB, superior to commercially available products (77.7 dB) (Fig. 11n) [197]. These innovations underscore the potential of ECNFAs to harmonize structural efficiency with spectral versatility, advancing their applicability in next-generation noise control systems for complex industrial and urban environments.

#### Electromagnetic Wave Absorption

The pervasive deployment of electromagnetic wave (EMW) technologies across radio/microwave (0.3–300 GHz), X-ray/γ-ray (> 30 PHz), and intermediate spectral bands has elevated electromagnetic pollution to a critical environmental challenge, now acknowledged as the fourth major pollutant after water, air, and noise contamination [198]. EMW absorption materials have emerged as indispensable solutions, which convert incident energy into thermal or electrical dissipation. Conventional coating- [199] or film-type absorbers [200, 201], despite their shape adaptability, suffer from interfacial delamination, low mechanical resilience, and excessive weight gain, rendering them unsuitable for dynamic operational environments. In contrast, structural absorbers [202, 203], exemplified by ECNFAs, unify EMW attenuation with load-bearing functionality through intrinsic material and geometric design. By leveraging hierarchical porosity, ECNFAs enable gradient impedance matching to achieve broadband microwave absorption with ultrathin profiles [204]. Concurrently, the nanofibers with high atomic numbers (Z) enhance the X-ray absorption efficiency via photoelectric dominance and multi-path scattering effect [205]. Furthermore, the synergy of ultralight frameworks and exceptional thermal insulation resolves the weight-durability trade-offs of traditional absorbers, while compressive stability ensures reliable operation even under extreme thermal and mechanical stress conditions. However, due to the relatively late start of the research, the current research indicates that ECNFAs are primarily applied in microwave absorption [206] and X-ray protection [98]. Therefore, the following discussion will focus on the design concepts and application innovations of ECNFAs in these areas of electromagnetic wave absorption.

**Fig. 12:**
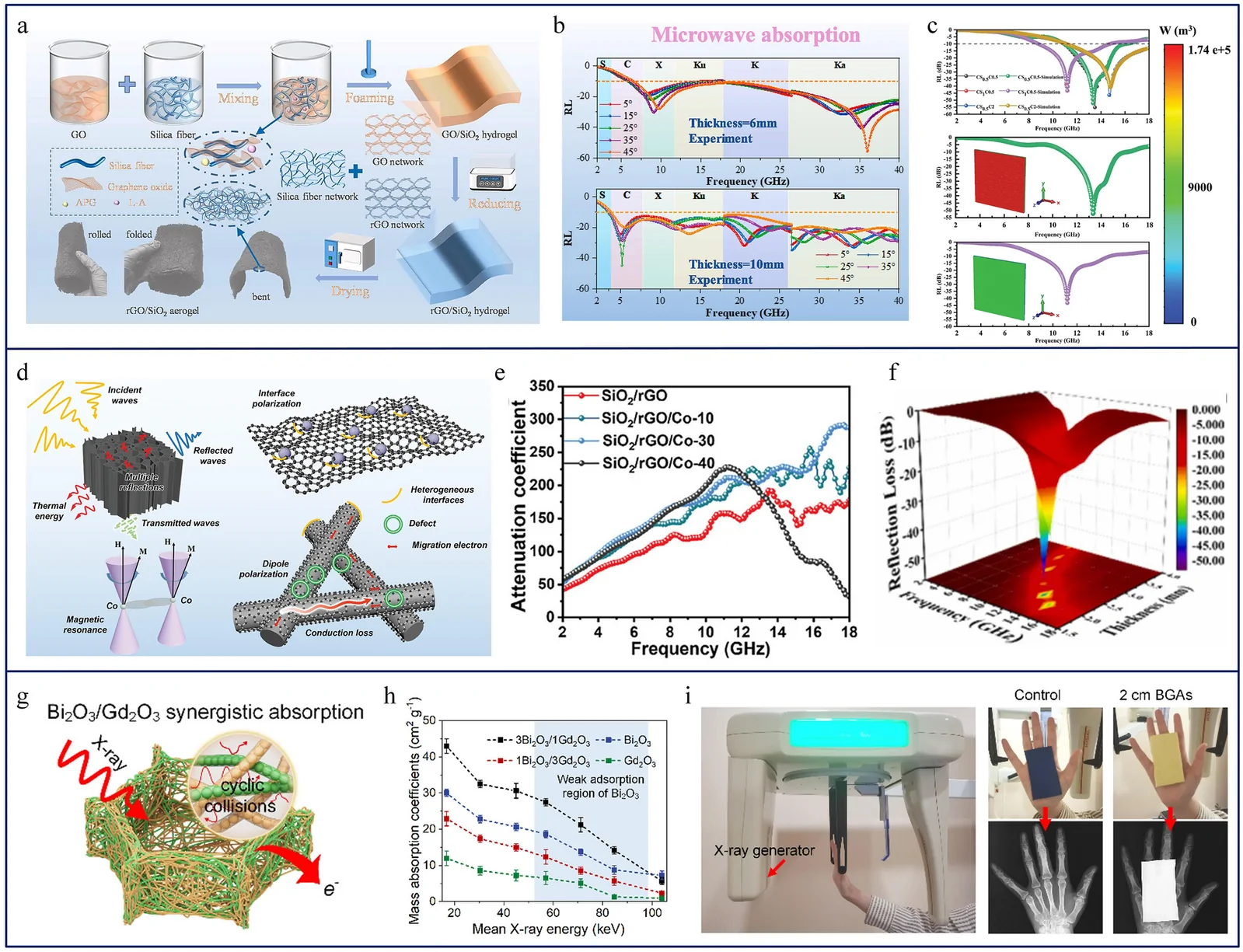
**a** Schematic diagram of preparation and **b** wide-angle EMW absorption effectivity of rGO/ECNFAs. Adapted with permission from Ref. [210]. Copyright 2024, Elsevier. **c** Schematic diagram of CSC nanofiber aerogels EMW absorption mechanism. Adapted with permission from Ref. [215]. Copyright 2024, Wiley–VCH. **d** Schematic diagram of EMW absorption mechanism and **e** EMW attenuation coefficient of Co–C/ECNFAs. Adapted with permission from Ref. [221]. Copyright 2024, Wiley–VCH. **f** EMW absorption effectivity of RGO/LZFO @SiO₂NFs aerogels. Adapted with permission from Ref. [222]. Copyright 2024, Elsevier. **g** Schematic diagram of X-ray absorption mechanism, **h** X-ray absorption effectivity, and **i** X-ray shielding physical image of Bi_2_O_3_/Gd_2_O_3_ nanofibrous aerogels. Adapted with permission from Ref. [225]. Copyright 2023, American Chemical Society

*Microwave Absorption* The pervasive use of microwaves (0.3–300 GHz) in modern technologies, such as 5G networks (2.4/28 GHz) [207], satellite communications (C-band: 4–8 GHz), and radar systems (X-band: 8–12 GHz) [54], has intensified electromagnetic interference (EMI) and environmental pollution, necessitating advanced microwave absorption materials. When microwaves interact with the surface of absorption materials, partial energy is reflected due to impedance mismatch, while the remainder penetrates and is dissipated as thermal or electrical energy via dielectric/magnetic loss mechanisms and undissipated waves transmit through the material. Therefore, to optimize absorption efficiency, two criteria must be met: (i) minimize surface reflection by achieving impedance matching and (ii) maximize intrinsic attenuation through tailored loss mechanisms [208]. According to transmission line theory, the reflection coefficient (*Γ*) at the air–material interface is governed by [209]:

8 $$\begin{array}{c}\Gamma =\frac{z-{z}_{0}}{z+{z}_{0}}\end{array}$$

where *Z* (material impedance) and *Z*_*0*_ (free-space impedance, ~ 377 Ω) need to match (*Z* ≈ *Z*_*0*_) to ensure minimized surface reflection. A representative example is the SiO_2_/rGO nanofiber aerogels (Fig. 12a), where the low dielectric constant of SiO_2_ materials reduces the composite’s effective permittivity, while the porous architecture of the aerogels lowers the effective refractive index, achieving near-perfect impedance matching (*Γ* ≈ 0). The design enables 6-mm-thick aerogels to achieve an ultra-wideband absorption bandwidth of 32.55 GHz, expanding to 37.21 GHz (range 2.79–40 GHz) at 10 mm thickness with a reflection loss of − 8 dB (Fig. 12b) [210].

Once optimal impedance matching is achieved, maximizing intrinsic attenuation emerges as a crucial step, primarily through dielectric loss and magnetic loss mechanisms [208]. Dielectric loss arises from conduction loss (resistive heating via free-electron oscillations in alternating electric fields) and relaxation polarization, which includes electron, ion, dipole orientation, and interfacial (space charge) polarization [211]. However, besides the semiconductor ceramics like silicon carbide (SiC) [212] and silicon boron carbonitride (SiBCN) [136, 213], which effectively utilize dielectric loss for microwave absorption, the majority of ceramics demonstrate poor attenuation capabilities which are attributed to their low dielectric activity and paramagnetic or diamagnetic behavior [214]. To overcome the limitation, integrating conductive phases into ceramic matrices has emerged as a proven strategy. For instance, incorporating carbon-based materials (e.g., CNTs [215], graphene [216]) into ECNFAs introduces polarization centers from structural defects and oxygen-functionalized sites, while heterogeneous interfaces (e.g., carbon/ceramic/air) induce space charge and interfacial polarization. A typical material is the C/SiO_2_@CNTs (CSC) nanofiber aerogels, wherein the conductive CNT network and carbon coating amplify relaxation polarization, ultimately achieving a minimum reflection loss of −55.16 dB at 3.4 mm thickness and a maximum effective absorption bandwidth of 8.5 GHz (range 6.695–15.195 GHz) at 4.5 mm (Fig. 12c) [215].

Magnetic loss serves as a pivotal mechanism for microwave dissipation, operating through pathways such as eddy current loss, hysteresis, domain wall resonance, natural resonance, and exchange resonance [217–220]. These processes amplify the material’s ability to convert microwave energy into heat, enhancing absorption efficiency. Integrating magnetic components into ECNFAs provides a strategic approach to enhance microwave absorption via magnetic loss. For instance, incorporating an appropriate quantity of the magnetic Co component into SiO_2_/rGO composite aerogel not only improved the permeability of the composite aerogel but also introduced magnetic loss (Fig. 12d) [221], which is more prominent in the high-frequency band, enabling the aerogels to exhibit better EMW absorption performance at a higher high-frequency band (Fig. 12e). In addition to the direct incorporation of magnetic particles into the aerogel framework to enhance magnetic loss, another effective approach for improving the magnetic loss of ECNFAs involves the construction of magnetic fibers, which can be achieved by integrating magnetic particles within the fiber matrix through electrospinning technology. By incorporating the magnetic spinel ferrite Li_0.35_Zn_0.3_Fe_2.35_O_4_ (LZFO) nanoparticles into SiO_2_ nanofibers, the magnetic LZFO@SiO_2_ composite fibers were yielded. These fibers were then hybridized with reduced graphene oxide (rGO) to fabricate RGO/LZFO@SiO_2_ nanofiber aerogels. The resultant ultrathin aerogel (2.2 mm) achieves exceptional performance, with a minimum reflection loss of −55.1 dB (Fig. 12f) and an effective absorption bandwidth of 4.3 GHz (11–16 GHz) [222]. The design not only advances ECNFAs’ applicability in microwave absorption but also demonstrates universal relevance for other ceramic systems, providing a reference for multifunctional composites that synergize magnetic and dielectric properties.

*X-ray Absorption* X-rays, characterized by high-frequency electromagnetic waves (0.01–1000 Å, 124 eV–1.24 MeV), are widely used in medical imaging and materials analysis due to their penetrating capability [223]. However, their ultrahigh energy nature necessitates effective shielding to mitigate cancer risks. Traditional lead (Pb) -based shield materials, while effective due to Pb’s high atomic number (*Z* = 82) and strong photoelectric/Compton absorption in the 20–100 keV range, face limitations due to toxicity and weight [224]. Alternatives like bismuth (Bi, *Z* = 83) and rare earth elements like gadolinium (Gd, *Z* = 64) offer comparable attenuation [225]. Meanwhile, Gd can enhance X-ray absorption in regions where lead and bismuth are less effective. Therefore, combining these elements can effectively enhance their X-ray absorption capabilities. Early strategies incorporated Gd_2_O_3_ nanosheets [98] or Bi_2_O_3_ nanoparticles [226] into polymer-based aerogels, utilizing synergistic effects for enhanced absorption. However, weak polymer-nanoparticle bonding limited durability. Xu et al. [205] developed flexible, self-winding Bi_2_O_3_/Gd_2_O_3_ nanofiber membranes (FJNMs), where continuous fibers avoided particle detachment. Despite the limitations, the planar geometry of the FJNMs restricted the propagation paths of X-rays, thereby decreasing their effectiveness. A significant breakthrough came in the form of isotropic Bi_2_O_3_/Gd_2_O_3_ aerogels, which drew inspiration from the hexagonal light traps found in natural leaves (Fig. 12g). The aerogels maximize photon capture through cyclic collisions that occur within the nanofiber arrays, achieving an impressive peak mass attenuation coefficient of 30.6 cm^2^ g^−1^ at an energy level of 45 keV (Fig. 12h) and efficient X-ray shielding in 16–90 keV, outperforming conventional Pb-based materials in terms of attenuation efficiency at the specific energy. The innovative design is capable of attenuating X-rays across a broad energy range from 16 to 90 keV. Moreover, by taking advantage of the ECNFAs, it ensures mechanical robustness, presenting a lightweight and durable alternative to traditional Pb-based shield materials (Fig. 12i) [225].

Current advancements in ECNFAs for microwave absorption predominantly rely on synergistic composites with carbon or magnetic phases, as pure ceramics face intrinsic limitations in dielectric/magnetic tunability. While SiC nanofiber aerogels exhibit good promise, their adoption is hindered by mechanical fragility and challenges in scalable electrospinning/3D stabilization, enabling SiC-based absorbers in the form of nanowires [227] or particle-reinforced aerogels [228, 229]. Future research must prioritize refining SiC nanofiber aerogel fabrication and diversifying ECNFAs’ compositions (e.g., borides, nitrides) to unlock broader EM absorption spectra. Concurrently, ECNFAs for X-ray protection remain nascent, with the current focus on bismuth/gadolinium oxides. Expanding this repertoire to include high-*Z* alternatives (e.g., tungsten, tantalum oxides) and enhancing mechanical resilience is critical to meet the durability demands of medical, aerospace, and industrial shielding applications. By bridging material innovation with structural engineering, ECNFAs can transcend current limitations, emerging as lightweight, multifunctional solutions for next-generation electromagnetic pollution management.

### Biomedical Field

**Fig. 13:**
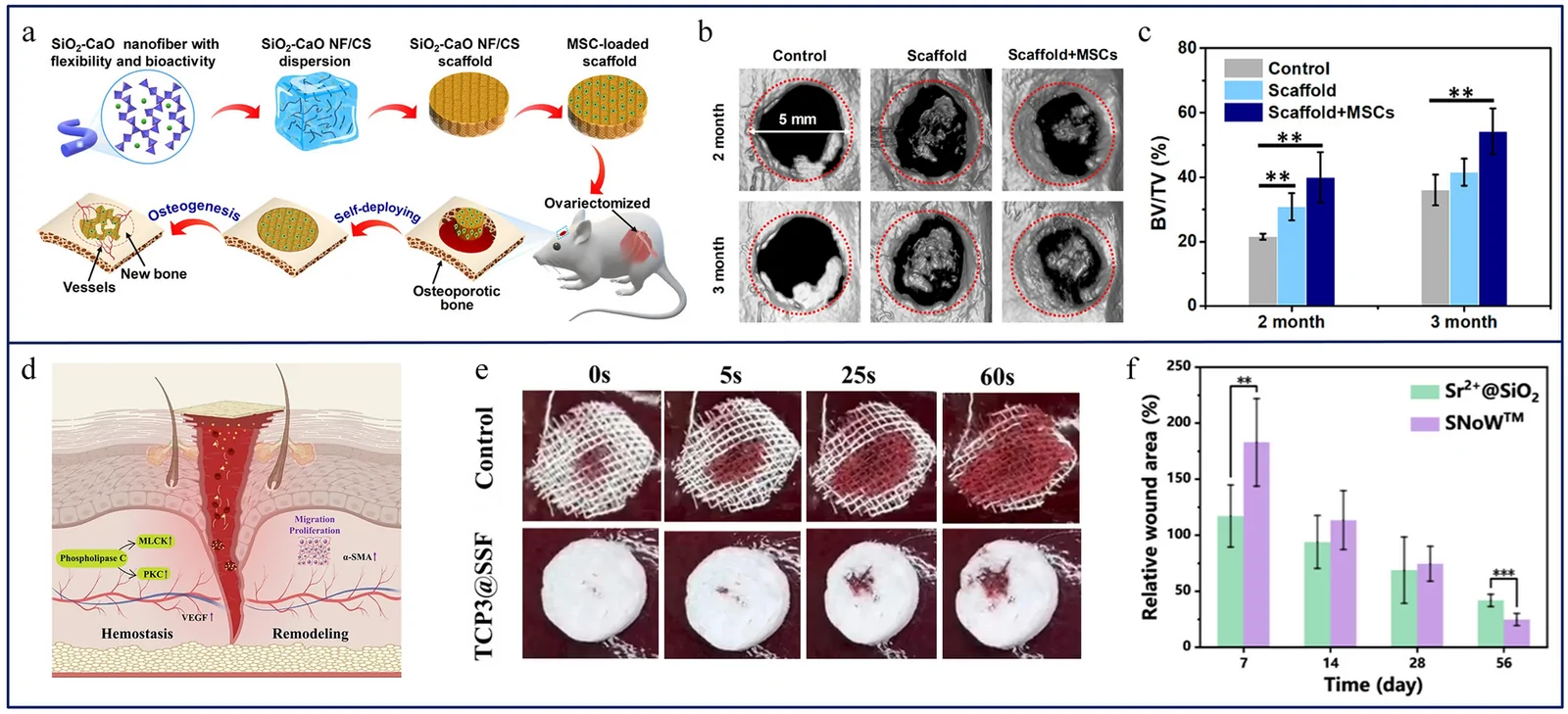
**a** Schematic diagram of fabrication, **b** micro-CT images, and **c** bone volume fraction of SiO_2_-CaO NF/CS scaffold. Adapted with permission from Ref. [239]. Copyright 2019, American Chemical Society. **d** Mechanism of skin regeneration and **e** the display of the hemostatic ability of TCPx@SSF aerogels. Adapted with permission from Ref. [241]. Copyright 2024, American Chemical Society. **f** Relative wound area changes over time after using M^2+^@SiO_2_ nanofiber aerogels. Adapted with permission from Ref. [242]. Copyright 2025, Elsevier

Bone tissue engineering relies on the synergistic triad of seed cells (e.g., mesenchymal stem cells, MSCs), growth factors, and scaffolds to regenerate functional bone tissue [230]. Ideal scaffold materials, including degradable organic materials or inorganic materials, exhibit biocompatibility, bioactivity, osteo-conductivity, and structural mimicry of native bone’s extracellular matrix (ECM) to support cell adhesion, proliferation, and mineralization [231–235]. Although natural and synthetic polymers, including polylactic acid/gelatin or polylactic acid-co-glycolic acid copolymer, are extensively employed as scaffold materials owing to their biodegradability and biocompatibility, their relatively limited osteo-inductivity necessitates the integration of inorganic components to mimic the ECM’s composition (60 wt%–70 wt% inorganic) and nanofibrous architecture. For instance, SiO_2_ nanofibers (NFs) or Si_3_N_4_ nanoparticles incorporated into polymer matrices (e.g., PLA/gel/SiO_2_ [33], PLGA/ Si_3_N_4_ [236]) enhance osteogenic differentiation via silicon ion release, which stimulates angiogenesis and cell proliferation [237]. With the development of tissue engineering technology, scaffolds made of inorganic fibers have a higher similarity to ECM [238], such as bioactive glass (SiO_2_-Na_2_O-CaO-P_2_O_5_) nanofibers, renowned for their osteoconductivity and ability to form hydroxyapatite layers, offer further promise in the field of scaffold materials. However, their brittleness hinders 3D nanofiber scaffold fabrication. Wang et al. synthesized the flexible 85SiO_2_-15CaO NFs (800 °C calcination) and reconstructed them into an elastic 3D aerogel (scaffold) using chitosan (CS) via freeze-drying (Fig. 13a) [239]. The resulting SiO_2_-CaO NF/CS scaffold integrated the bioactivity of Ca^2+^-releasing nanofibers with CS’s shape-memory elasticity, achieving a compression modulus of approximately 12 MPa and conformability to irregular defects. In vivo, cranial defect studies demonstrated enhanced osteogenesis and angiogenesis (Fig. 13b, c), attributed to MSC-secreted exosomes/growth factors and sustained Ca^2+^ release. The design exemplifies how ECNFAs overcome traditional limitations, merging mechanical resilience (elasticity, ~ 80% strain recovery) with bioactivity to advance next-generation bone grafts.

Effective hemorrhage control and wound healing remain critical challenges in clinical practice, as conventional hemostats [240] (e.g., gauze, sponges, hydrogels) face limitations such as poor exudate absorption, limited penetration depth, and uncontrolled gelation rates. ECNFAs address these challenges through their ultrahigh porosity, compressible elasticity, and tailorable bioactivity. For instance, tricalcium phosphate (TCP)-functionalized SiO_2_ nanofiber aerogels (TCPx@SSF) achieved rapid hemostasis (32.3 ± 2.5 s) via Ca^2+^-mediated platelet activation and fibrin cross-linking. Post-hemostasis, sustained release of Si^4+^ and Ca^2+^ ions promoted angiogenesis (via VEGF/α-SMA upregulation) and keratinocyte migration, enabling 95.5% ± 7% wound closure within 14 days (Fig. 13d, e) [241]. Similarly, divalent metal ion (M^2+^ = Ca, Mg, Sr)-loaded SiO_2_ aerogels (M^2+^@SiO_2_) facilitated liver soft tissue regeneration by providing a porous scaffold for endothelial cell infiltration and collagen deposition (Fig. 13f) [242].

Surprisingly, in addition to bone tissue engineering, as well as hemostasis and soft tissue repair, ECNFAs also excel as drug delivery platforms due to their hierarchical pore networks and high specific surface area. Drug release materials are designed to encapsulate or disperse therapeutic agents, enabling controlled, sustained release to prolong efficacy, reduce dosing frequency, and minimize toxicity. Unlike conventional systems (e.g., nanoparticles [243, 244], hydrothermally synthesized nanowires [245–247]), ECNFAs enable precise regulation of drug release kinetics (e.g., zero-order, burst, or stimuli-responsive release) through pore size modulation, surface functionalization, and ionic doping. For instance, a ZnO-SiO_2_/chitosan@aspirin (ZnO-SiO_2_/CS@ASA) scaffold demonstrated dual functionality as a bone repair scaffold and anti-inflammatory drug carrier, sustaining ASA release over 14 days while inducing macrophage polarization to the pro-regenerative M2 phenotype, thereby fostering an anti-inflammatory microenvironment [248]. However, challenges persist in achieving high drug encapsulation efficiency and predictable biodegradation rates, as current ECNFAs-drug systems remain in early developmental stages. Limited understanding of pore–drug interactions and insufficient in vivo validation hinder clinical translation. Therefore, further research is needed for the application of ECNFAs in drug delivery.

In conclusion, ECNFAs exhibit significant potential in bone tissue engineering due to their extracellular matrix (ECM)-mimetic architecture, hierarchical porosity, and tunable bioactivity. However, strategies to enhance biodegradability, such as polymer doping (e.g., chitosan, PLGA), often compromise mechanical compatibility with native bone (e.g., elastic modulus mismatch, reduced compressive strength), limiting clinical translation. Biodegradable bioceramics (e.g., bioactive glass [238], calcium phosphate (CaP) [249]) address these limitations but face challenges in the scalable fabrication of 3D ECNFAs due to inherent brittleness. Beyond bone repair, ECNFAs also have extensive potential applications in wound healing and soft tissue regeneration. The high surface area and tailorable surface chemistry could enable the spatiotemporal release of growth factors or immunomodulatory agents, while their nanofibrous topography may guide cell alignment in soft tissues. However, challenges such as biodegradation rate control, long-term in vivo biocompatibility, and drug delivery control require systematic investigation.

### Other

Beyond their established applications in thermal insulation, gas purification, water treatment, noise management, electromagnetic wave absorption, and biomedicine, ECNFAs are being investigated for novel applications like industrial ammonia production [32, 250] and osmotic power generation [251], thereby expanding the potential applications of ECNFAs. In industrial ammonia production, the Haber–Bosch process relies on Fe/Ru catalysts and operates under extremely harsh conditions (400–550 °C and 15–25 MPa) [252–254], which suffers from excessive energy consumption. In contrast, the electrocatalytic nitrogen reduction reaction (E-NRR), which uses water as a proton source, has emerged as a more sustainable alternative. However, the efficiency of the method hinges on the development of efficient catalysts [255]. TiO_2_ nanofiber aerogels, characterized by their large surface area, hierarchical porosity, and adjustable electronic properties, present a revolutionary solution. Oxygen vacancies can be introduced through methods such as treatment with lithium metal in an Ar atmosphere [32] or NaBH_4_-mediated hydrothermal reduction [250] to lower the N₂ activation barrier, achieving an NH_3_ yield of 4.81 × 10^–10^ mol s^−1^ cm^−2^ and a Faradaic efficiency of 20.3% at -0.55 V (Fig. 13g-j). Another promising application of ECNFAs in the energy sector is osmotic energy harvesting, which is achieved by ion-selective transport between two solutions with different salt concentrations [255]. To achieve rapid and selective transport of ions, Zhang et al. [251] designed biomimetic SiO_2_/PANI nanofluidic cable aerogels (NMIMs) inspired by electric eel ion channels (Fig. 13k). The NMIMs’ interconnected pore structure selectively enhances Cl⁻ transport, achieving energy densities of 30.7 W m^−2^ (seawater/freshwater, 0.5/0.01 M) and 133.2 W m^−2^ (Chaka Salt Lake/freshwater, 0.5/0.01 M) (Fig. 13l). The bioinspired strategy bridges nanoscale ion dynamics with macroscale energy conversion, offering scalable solutions for blue energy utilization.

## Conclusions and Outlook

In summary, this review systematically examines the preparation of ECNFs via electrospinning technology and their intrinsic properties, establishing a foundational framework for the development of ECNFAs. The synthesis of ECNFAs through methods such as freeze-drying, stacking, foaming, and direct spinning is comprehensively analyzed, with ECNFs identified as the core structural building blocks. The structural regulation of these aerogels, particularly the optimization of mechanical properties (such as compressive resilience, elastic modulus), is emphasized, alongside multi-scale strategies to enhance performance, including building blocks optimization, bonding points construction, and nanofiber aggregate structures design. Owing to their ultrahigh porosity (> 90%), interconnected pore networks, and functionalization versatility, ECNFAs demonstrate significant potential in thermal insulation, environmental remediation (such as pollutant adsorption, catalysis), and tissue engineering such as bone regeneration scaffolds. While extensive research has been conducted on ECNFAs and significant progress has been made, there remains a substantial gap between their current performance and practical applications. To advance the future development of ECNFAs, the following section outlines some of the major challenges and future directions for improvement:

(1) Expanding basic research beyond functional applications. Currently, research on ECNFAs is limited. Therefore, the foundational theory of ECNFAs, whether in terms of structure, mechanical properties, or application performance, is still incomplete. Future research should focus on establishing a comprehensive theoretical framework for ECNFAs by studying spinning methods, the intrinsic acoustic, optical, electrical, thermal, and magnetic properties of the fibers, and the relationships between preparation methods and structure, as well as the structure–property relationships. It is hoped that with the aid of artificial intelligence, the theoretical framework will enable optimal design from chemical raw materials to the functional applications of ECNFA-based materials.

(2) Expand material systems and research scope. Currently, both the ECNFs used as building blocks and ECNFAs are limited to a narrow range of ceramic materials. Future research should focus on expanding the variety of ECNFs that can be used to fabricate ECNFAs and broadening their application areas. For instance, by developing more advanced spinning techniques, it is possible to produce a wider range of flexible non-oxide ceramic fibers. This would allow for the expansion of ECNFAs into new domains such as energy and smart devices.

(3) Addressing challenges in large-scale production. The preparation of ceramic nanofiber aerogels, involving electrospun ceramic nanofibers and fibrous three-dimensional assembly, is complex and time-consuming compared to that of traditional aerogels, resulting in serious costs and energy consumption. Nevertheless, the superior mechanical properties exhibited by ECNFAs significantly surpass those of conventional aerogels, enabling their widespread adoption in intricate service environments such as air-to-space metamorphic vehicles and flexible wearable battery technologies, where their advantages greatly outweigh any associated cost increases. Nevertheless, the scalability of manufacturing ECNFAs remains a significant bottleneck: high-voltage electrospinning technology is limited by needle tip interference, which restricts production efficiency. Although butterfly nozzles and needleless electrospinning have increased output, subsequent steps such as restructuring, drying, and calcination are still time-consuming, further hindering industrial-scale manufacturing. Recently, advancements in direct spinning methods (e.g., blow-spinning and 3D reactive spinning) have shown great potential for rapid aerogel formation, offering promising avenues for large-scale production. During the process, continuous innovation in bulk manufacturing techniques will be key to accelerating the commercialization of ECNFAs.

As an advanced material that combines the structural advantages of ceramic nanofibers with the unique properties of aerogels, ECNFAs hold broad application prospects in various fields of advanced technology. However, to fully realize their potential, challenges in optimizing mechanical properties, achieving multifunctionality, and scalable manufacturing must be overcome. With ongoing research advancements and breakthroughs in design strategies, processing techniques, and application development, ECNFAs are poised to make significant progress, paving the way for transformative innovations in materials science.
